# Cancer cells are highly susceptible to accumulation of templated insertions linked to MMBIR

**DOI:** 10.1093/nar/gkab685

**Published:** 2021-08-11

**Authors:** Beth Osia, Thamer Alsulaiman, Tyler Jackson, Juraj Kramara, Suely Oliveira, Anna Malkova

**Affiliations:** Department of Biology, University of Iowa, Iowa City, IA 52245, USA; Department of Computer Science, University of Iowa, Iowa City, IA 52245, USA; Department of Biology, University of Iowa, Iowa City, IA 52245, USA; Department of Biology, University of Iowa, Iowa City, IA 52245, USA; Department of Computer Science, University of Iowa, Iowa City, IA 52245, USA; Department of Biology, University of Iowa, Iowa City, IA 52245, USA

## Abstract

Microhomology-mediated break-induced replication (MMBIR) is a DNA repair pathway initiated by polymerase template switching at microhomology, which can produce templated insertions that initiate chromosomal rearrangements leading to neurological and metabolic diseases, and promote complex genomic rearrangements (CGRs) found in cancer. Yet, how often templated insertions accumulate from processes like MMBIR in genomes is poorly understood due to difficulty in directly identifying these events by whole genome sequencing (WGS). Here, by using our newly developed MMBSearch software, we directly detect such templated insertions (MMB-TIs) in human genomes and report substantial differences in frequency and complexity of MMB-TI events between normal and cancer cells. Through analysis of 71 cancer genomes from The Cancer Genome Atlas (TCGA), we observed that MMB-TIs readily accumulate *de novo* across several cancer types, with particularly high accumulation in some breast and lung cancers. By contrast, MMB-TIs appear only as germline variants in normal human fibroblast cells, and do not accumulate as *de novo* somatic mutations. Finally, we performed WGS on a lung adenocarcinoma patient case and confirmed MMB-TI-initiated chromosome fusions that disrupted potential tumor suppressors and induced chromothripsis-like CGRs. Based on our findings we propose that MMB-TIs represent a trigger for widespread genomic instability and tumor evolution.

## INTRODUCTION

Massive chromosomal rearrangements are hallmarks of cancer and can promote a number of other diseases as well. The discovery of chromothripsis, which is associated with various types of cancer, demonstrated that chromosomal rearrangements can be highly complex, and that they are often induced by a single, catastrophic event (1–5). This represented a significant shift in our understanding of chromosome instability, which had previously been believed to represent accumulation of individual, small genome changes over time. To explain the nature of the initial event triggering such catastrophes, two mechanisms were proposed. First, the massive shattering of a chromosome followed by random stitching together of fragments by non-homologous end-joining (NHEJ) resulting in chromothriptic chromosomes was supported by analysis of cancer genomes by Illumina sequencing (1,3,5). Alternatively, it has been proposed that chromothripsis may represent abnormal DNA synthesis that proceeds by multiple rounds of template switching to produce complex genome rearrangements (CGRs) and sometimes copy number gains (4,6–8). The latter mechanism has been also called ‘chromoanasynthesis’ in order to emphasize its DNA-copying nature, and it was originally described for CGRs associated with several congenital neurological disorders, which revealed that the genetic rearrangements at CGR break sites are highly complex (9–12). Specifically, Sanger sequencing analysis of the segmental duplications thought to cause Pelizaeus–Merzbacher disease (PMD) identified complex combinations of break points, which presented evidence of template switching at microhomologies that led to copy number gains from duplications up to quadruplications (10,12). The authors proposed that this mechanism, named microhomology-mediated break-induced replication (MMBIR) (13–15), underlies a number of neurological disorders, and represents an alternative to NHEJ in triggering chromothripsis in cancer (7). A fraction of MMBIR events contained templated insertions at the place where MMBIR was initiated, and these insertions were proposed to reflect the initial step or precursors of complex genomic rearrangements (including CNVs) associated with MMBIR (10,11,16). Initial Illumina sequencing of chromothriptic oncogenomes did not identify templated insertions at breakpoints indicative of MMBIR (1,3,5). However, a recent study of chromothriptic genomes selected from over 2500 cancers, which also used more sophisticated analysis methods, occasionally revealed templated insertions that could be explained by MMBIR at some chromothriptic junctions (17).

Despite this recent progress, the frequency of MMBIR-initiating **t**emplated **i**nsertions (MMB-TIs) and how significantly they may contribute to genomic instability in cancer remain poorly understood. Currently, our knowledge of MMB-TIs is based on characterization of the junctions of CGRs or copy number variations (CNVs) (8,10–12,16–20). This limits our understanding to only those MMBIR events that result in CGRs, and even in these cases, some MMB-TI events are missed due to alignment problems. It is likely that MMB-TIs may occur more frequently but result in small-scale genetic lesions that are not detectable using current approaches. To advance our understanding of the contribution of MMB-TIs to genetic stability and genome evolution, a forward approach with the resolution to detect individual MMB-TI events that are not defined by whether they produce CGRs is required.

Previously, we modeled MMB-TI events in a yeast experimental system, where we observed that that MMBIR can be initiated by the interruption of break-induced replication (BIR) (21), provoked by the absence of Pif1 helicase which is required for processive BIR replication, reviewed in (22,23). In these studies we observed template switching events at sites of microhomology that led to the formation of short insertions copied from a nearby template (within 100 bp from the position of insertion) and that were flanked by microhomology (21). These events were highly similar to MMB-TIs proposed to initiate MMBIR events in humans (8,10,11,16).

**Figure 1.**
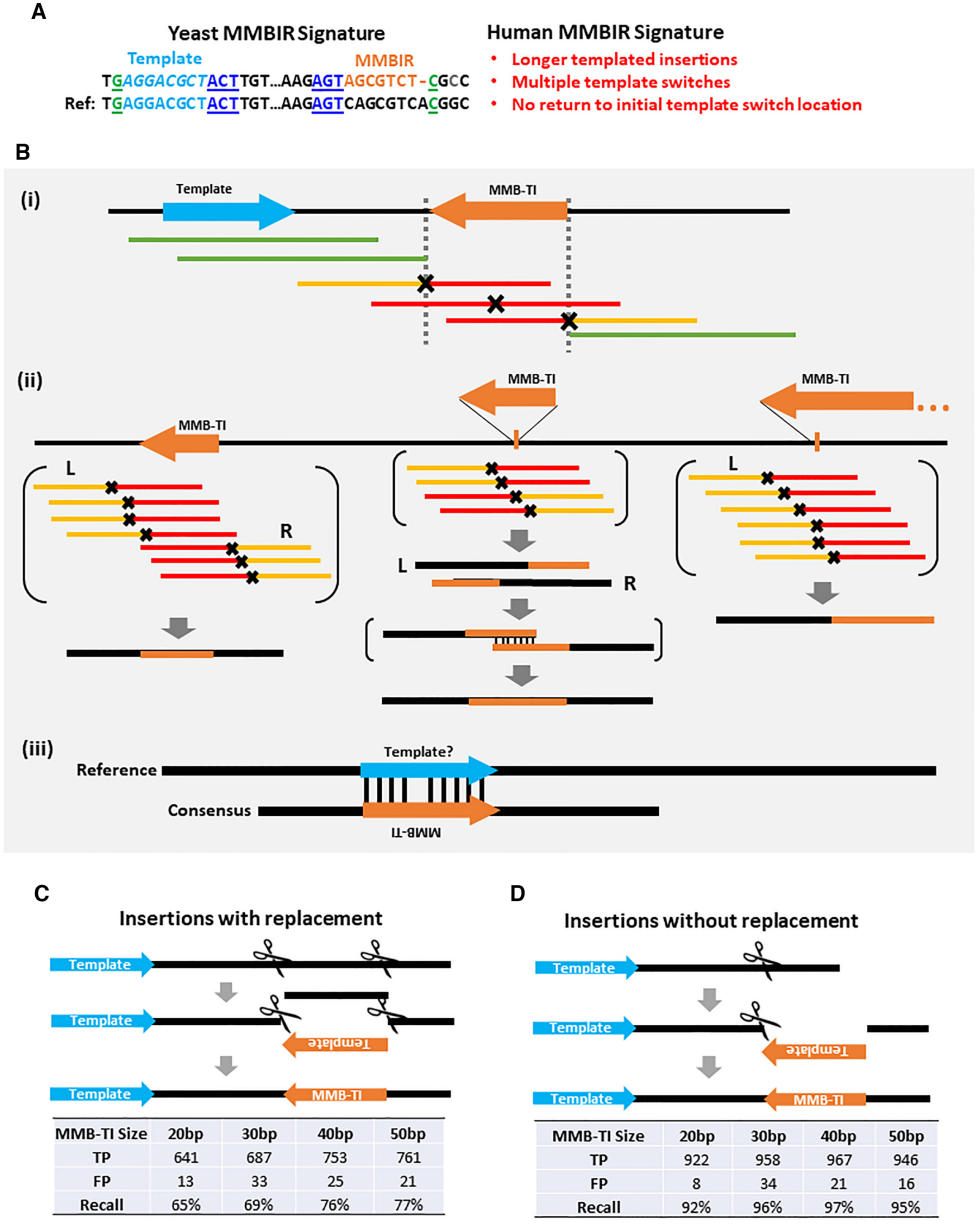
MMBSearch is a novel tool to detect MMB-TI events in NGS reads. (**A**) Signatures of MMBIR from yeast and from human genomes that produce MMB-TIs. (Left) Top line: The outcome (signature) of MMB-TIs from studies in yeast (21) is an insertion (orange text) located in proximity and inverted orientation to its complementary template (light blue text) and flanked by microhomologies at the junctions (green and dark blue underlined text). Bottom line: The original sequence (Ref). (Right) Features of MMB-TIs in humans (red text) based on studies of CNVs in congenital diseases (8,10–12,16–20). (**B**) The MMBSearch tool identifies MMB-TIs based on the signatures described in (A): (i) reads containing MMB-TIs (red), which do not align to a reference genome are collected and re-aligned as half-reads. (ii) Reads where the first half (yellow) aligns while the second half (red) does not are collected and clustered by position of the aligned halves. The aligned read halves serve as anchors, and whole reads anchored on the same side (right – R or left – L) are used to create consensus sequences. If R and L consensuses overlap by position and are discordant (middle example), they are aligned to each other to create the full accurate consensus, which is compared back to the reference genome to identify the full MMB-TI sequence. (iii) The reverse-complement of the MMB-TI is aligned to the reference genome within 100 bp from the insertion to identify a possible template. (**C**) (top) Schematic for creation of synthetic insertions by replacing sequence with the reverse complement of a template from 80bp upstream (5′ of the insertion). (bottom) Number of true positives (TP), number of false positives (FP) and recall (TP/total) called by MMBSearch from processing of synthetic reads generated from Chromosome 17 with 993 synthetic insertions sized 20–50 bp. (**D**) (top) Schematic for creation of synthetic insertions by appending the reverse complement of a template from 80bp upstream (bottom). Analysis for the reads in top analyzed similarly to (C).

Here, we have combined the MMB-TI signature observed in yeast with features of templated insertions that were observed in association with human MMBIR events (e.g. increased sizes of templated insertions, involvement of several templates, and several other features reflecting higher complexity of MMB-TI events described as being associated with congenital diseases and cancer) (Figure 1A) to create a new software tool called MMBSearch, which uses a highly sensitive mapping strategy to identify templated insertions from whole-genome sequencing (WGS) reads irrespectively of whether they are associated with CNVs or CGRs. Using our MMBSearch software, we report the first genome-wide characterization of MMB-TIs in normal and cancer human cells. We observed accumulation of MMB-TI events across several types of human cancers. By contrast, MMB-TI events in normal human skin fibroblasts were present only as germline events and did not accumulate in somatic cells. Finally, by performing WGS on a non-small-cell lung cancer patient case we identified tumor-specific MMB-TI events that initiated a chromothripsis-like pattern of CGRs. Our data demonstrate that MMB-TI accumulation represents a specific signature of cancer cells, while MMBSearch is a powerful tool that can be used effectively in multiple cell contexts to analyze the frequency of MMB-TIs and the associated genetic outcomes.

## MATERIALS AND METHODS

### Read preprocessing and the MMBSearch tool

MMBSearch was used with either raw reads files or reads extracted from an alignment (BAM) file. Samtools (version 1.9) was used to extract unmapped reads from BAM files via the alignment flag, while soft-clipped reads and reads with indels were extracted from gapped alignments (those aligned by BWA-MEM (24) by selecting reads containing ‘I’, ‘D’ or ‘S’, in their CIGAR string. For ‘preprocessing’ of reads to be analyzed by MMBSearch, PRINSEQ (25) was used to perform low-complexity filtering via the DUST algorithm (threshold = 7). Trimmomatic (version 0.38) (26) was then used to perform adapter trimming with the Truseq3 Single-end adapter set and to perform trimming of low quality bases and N bases from read ends using the following parameters: LEADING:3 TRAILING:3 SLIDINGWINDOW:4:15 MINLEN:36.

After preprocessing, MMBSearch aligns reads using BWA-backtrack and an error threshold of 4. MMBSearch then recovers all unmapped reads (those with more than four differences from the reference) and splits them in half. The half-reads are again aligned to the reference by BWA-backtrack with the same error threshold, and the reads with one mapping and one non-mapping half are accepted for clustering. Clusters that meet the user-specified threshold of reads are analyzed by their anchoring side (mapping half-read for each read in the cluster). Clusters that anchor all left, all right, or left and then right are accepted, and consensus sequences are called for the anchored reads. The two consensuses that form from left-then-right clusters are locally aligned to each other to correct any positional errors. The full consensus contigs are then compared back to the reference sequence to determine the sequence of a candidate MMB-TI region (insertion or breakpoint). The sequence of the MMB-TI region is then used to search for a nearby template (within 100 bp). MMBSearch outputs files containing the consensus contigs, full clusters, and called MMB-TI events (a list of insertions, reference locations, and alignments of each insertion to its template). MMBSearch parameters used in this study for initial quantification of MMB-TI events and parameters for in-depth analysis are listed below (also see Supplementary Figure S1A). For paired samples analyzed by MMBSearch, results files can be compared to one another to identify MMBSearch calls that are common between the two samples or unique to one sample or the other (see Supplementary Figure S1B).

The source code for MMBSearch and a full tutorial containing instructions for pre-processing WGS reads, instructions for installing MMBSearch and running MMBSearch in a high performance computing cluster environment, basic post-processing instructions (comparison of results between two samples, see Supplementary Figure S1B), and descriptions of results file entries are available from https://github.com/malkovalab/MMBSearch.

Reads derived from clonal skin fibroblasts (27) (NCBI accession: PRJNA336369; dbGaP: phs001182), and reads derived from dbGaP IB lung adenocarcinoma (NCBI accession: PRJNA157939; dbGaP: phs000488) were analyzed with respect to human genome reference version GRCh37/hg19 to maintain positions with previous studies, while all other samples were analyzed with respect to GRCh38/hg38 for posterity.

Fibroblast data sets were analyzed using the following MMBSearch configuration parameters: For the initial (quantitative) analysis of all events, minimum MMB-TI event length was set to 10 bp with a minimum 80% identity between the MMB-TI and its reference template. Secondary (detailed) analysis to find more complex MMB-TI events and to eliminate interference from shorter microsatellites was achieved by setting the minimum MMB-TI length to 25 bp, with a minimum of 40% identity between the MMB-TI event and the template. For both analyses, minimum cluster size was set to 10 reads. Cluster size was reduced to 3 reads for the sample used for comparison to cancer genomes (Supplementary Figure S8). Similarly, for both lung adenocarcinoma datasets and all TCGA datasets, minimum MMB-TI event length was set to 10 bp with a minimum identity of 80% between MMB-TI and template for quantitative analysis. The two lung adenocarcinoma samples analyzed in-depth underwent secondary (detailed) analysis, allowing a minimum of 20 or 25 bp depending on read length (100 and 150 bp respectively) with 40% identity between MMB-TI and template. For all cancer datasets, minimum cluster size was set to 3 reads (All other MMBSearch configuration parameters were left as default unless otherwise specified). The number of reads of evidence for MMB-TI events and structural variant (SV) junctions were used to infer clonality by dividing the number of MMB-TI or SV event reads by the reported average mappable coverage of the genome. Events with read counts <30% of the average mappable coverage were considered to be sub-clonal.

Fibroblast and lung adenocarcinoma datasets that were analyzed in-depth were manually inspected to exclude false positive calls and microsatellite calls, and had positions of germline (Fibroblasts) and sub-clonal to clonal (Lung adenocarcinoma) MMB-TIs confirmed using BLAT (28) to exclude ambiguous calls (See Supplementary Figure S1C for details and examples of all manually excluded results) and to help resolve more complex multi-templated MMB-TI events. BLAST (29) was used to query NCBI’s Nucleotide collection to search for previously published germline insertions.

For manually inspected junctions with resolvable templates, microhomology was counted by comparing the insertion junctions identified by MMBSearch in sequencing reads to the reference-derived complement to the insertion (template) and its flanking sequence. Microhomology was defined as uninterrupted matching bases at the junction. Likewise, the allowance of a single mismatch or gap that interrupts the matching bases at a junction and results in at least one additional complementary base is referred to as ‘microhomeology.’

Read aggregate analysis of lung adenocarcinoma samples BO13 and BO14 to determine changes in copy number was performed using CLC Genomics Workbench 20. Ploidy was inferred by comparing read coverage in regions with apparent copy number changes to estimated average coverage for the genome.

### MMBSearch sensitivity testing

We generated 993 artificial MMB-TIs of various sizes (20–50 bp) in GRCh37/hg19 Chromosome 17 at regular intervals, excluding regions of Chromosome 17 that contain N bases. Using ART (version: MountRainier-2016-06-05) (30) we then simulated paired-end 125 bp Illumina reads with an average insert size of 500 bp with a standard deviation of 20, to an average depth of 30×. These reads were analyzed with MMBSearch with search parameters set to a minimum MMB-TI length of 6 bp with a minimum of three reads to form a cluster. The results output was then analyzed for true positives (TP) by comparing MMBSearch MMB-TI calls to the list of artificial MMB-TI insertions. Remaining calls or duplicate calls were considered to be false positives (FP). The number of false negatives (FN) was determined by subtracting TP from the total events inserted. Recall was calculated with the following formula: TP/(TP + FN).

### Molecular biology methods

Genomic DNA was prepared from tumor and non-tumor tissues frozen in OCT medium following tumor resection using the Qiagen blood and Cell culture DNA Mini Kit (Cat no. 13323). Library preparation was performed for whole genome sequencing using the TruSeq DNA PCR-Free (350) kit (protocol: TruSeq DNA PCR-Free Sample Preparation Guide, Part # 15036187 Rev. A). Sequencing was performed on the Illumina NovaSeq6000 S4 (2 × 150 bp) sequencing platform. Tumor (BO13-UIBB-1654) tissue was sequenced to an estimated average coverage of 51×, and non-tumor (BO14-UIBB-1654) tissue was sequenced to an estimated average coverage of 65.5×.

GM1604 human fetal lung fibroblast cells were cultured in Dulbecco's modified Eagle's medium (DMEM, Life Technologies) supplemented with 10% fetal bovine serum (FBS, Gibco) and 100 units/ml of penicillin/streptomycin solution (Life Technologies). Cells were incubated at 37°C and 5% CO_2_ in a humidified atmosphere. GM1604 clones were obtained by expanding cell colonies originating from a single cell. Genomic DNA from the expanded human fetal lung fibroblast clones was purified using the Qiagen blood and Cell culture DNA Mini Kit. Library preparation was performed for whole genome sequencing using the TruSeq DNA PCR-Free (350) kit (protocol: TruSeq DNA PCR-Free Sample Preparation Guide, Part # 15036187 Rev. A). Sequencing was performed on the Illumina NovaSeq6000 S4 (2 × 150 bp) sequencing platform to achieve an estimated average coverage of 26–32× MMB-TIs and associated rearrangement junctions found in Lung tumor (BO13-UIBB-1654) were confirmed by PCR. Forward and reverse primers were designed to the boundaries of each junction (See Supplementary Data S20 for all primers used), and PCR was performed using Phusion High-fidelity DNA Polymerase (NEB Cat no. M0530S). PCR products of junctions containing MMB-TI events were sequenced by Sanger sequencing to confirm the presence of the MMB-TI within the rearranged allele. All PCRs were also performed with non-tumor DNA from the same patient (BO14-UIBB-1654) to confirm tumor specificity of the rearrangement junctions.

## RESULTS

### MMBSearch identifies insertions with nearby templates in NGS datasets

To identify MMB-TI events genome wide, we developed a computational search tool, MMBSearch, that uses an MMB-TI signature determined from our studies in yeast (21) as a foundation and expands upon the signature to also include potentially longer events, events with multiple instances of template switching, and events that do not return to the same location (Figure 1A). Expanding the signature to include these features makes MMBSearch capable of detecting MMB-TI events similar to those described in association with human diseases (8,10,11,16,17,19,20). Specifically, MMBSearch identifies small templated insertions (10 bp at minimum and the full length of the sequencing reads at maximum) that represent inverted copies of their templates located within 100 bp from the insertion. Because standard genomics pipelines often exclude reads containing insertions comparable in length to the read size, we engineered MMBSearch to call only sequencing reads that were excluded by initial alignment, and to use a split-read alignment strategy to create clusters of candidate MMB-TI reads based on the positions of their reference-matching half-read anchors (Figure 1Bi) (see Materials and Methods). The resulting clusters of reads are used to create an accurate consensus (a single sequence built from the agreement of all reads in a given cluster) from which the exact boundaries of the insertion can be determined and used to search for nearby inverse-complementary sequence representing the template (Figure 1Bii and iii).

We tested the sensitivity of MMBSearch using a set of artificially created human genomes that each contained 993 MMB-TIs of varying lengths (from 20 to 50 bp) on human chromosome 17 (see Materials and Methods). Insertions that both replaced or did not replace the sequence at the insertion site were included (Figure 1C, D). MMBSearch analysis of artificial Illumina sequencing reads generated from these test genomes demonstrated recall (sensitivity) of 92–97% for insertions without replacement (Figure 1D) and recall of 65–77% for insertions with replacement (Figure 1C). False-positive calls were low in both cases (1–3%) (Figure 1C, D) (see Supplementary text for details). Thus, MMBSearch accurately calls MMB-TI events in human genome datasets.

### Normal skin fibroblasts contain MMB-TIs only as germline variants

To determine whether we could identify MMB-TI events in normal somatic cells, we obtained sequencing reads originally prepared from clonal fibroblast lineages by (27). In this study, the authors assessed the frequency of mutations accumulated in an individual's lifetime by sequencing clonal lineages derived from several locations on the forearms and hips of two individuals. Importantly, the ten different cell lineages in this study all represented independent cases of mutation accumulation. The authors demonstrated that, in each of these lineages, both base substitutions and chromosomal rearrangements accumulated at frequencies that were as high as those associated with some cancers (up to hundreds of mutations per year), prompting the hypothesis that MMB-TI mutations may be also common and/or accumulate in these fibroblast lineages as well.

To test our hypothesis, we analyzed sequencing reads from all 10 clonal fibroblast cell lineages and their matched blood samples (6 clones from individual D1 in the study and four clones from individual D2; for full description of analysis parameters, see Supplementary text and Supplementary Figure S1). Although each clone produced over 1000 MMBSearch calls, we observed that the majority (74–93%) of all MMBSearch calls among all 10 fibroblast clones were germline variants (Supplementary Data S1, Supplementary Figure S2; Supplementary Data S2, S3). Of the remaining 7–26% of MMB-TI calls, which we identified as fibroblast-specific events, manual curation determined that all calls but one were either present among multiple fibroblast clones, represented microsatellite alterations, or were determined to be false-positive outcomes (Supplementary Data S1–S4). Thus, we found no evidence of MMB-TI accumulation in fibroblasts with age, which represents an intriguing difference between MMB-TI mutational events and other mutations that accumulated at high frequency in aging fibroblasts (27).

**Figure 2.**
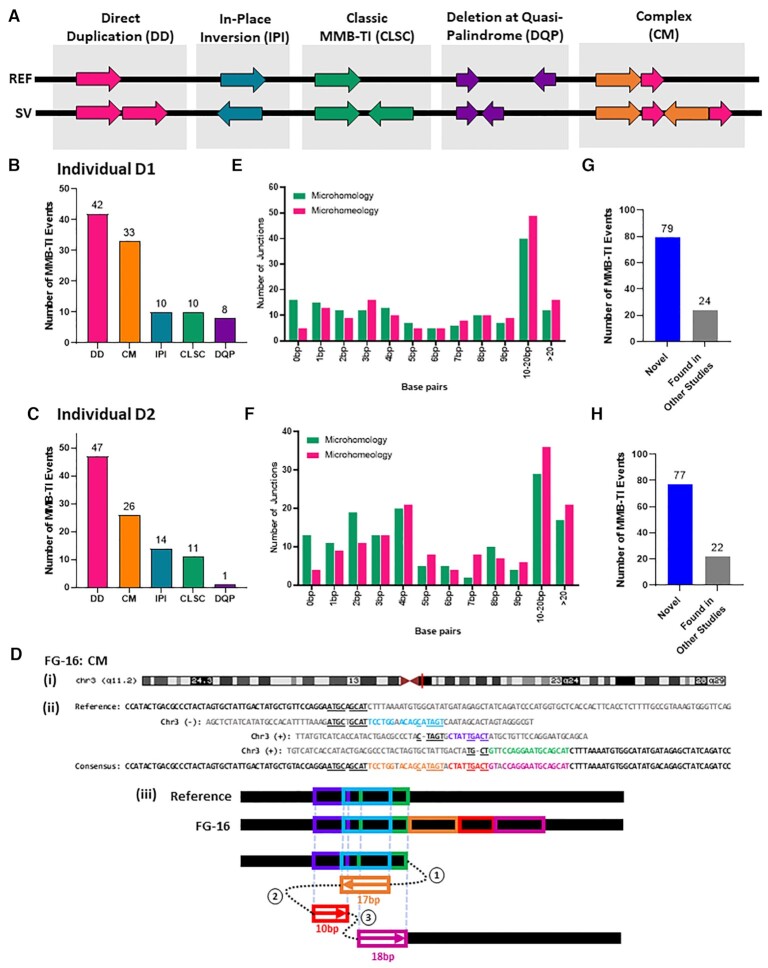
MMB-TIs are found as germline variants in human fibroblasts. (**A**) Schematic of different classes of MMB-TI events. REF = reference; SV = MMB-TI event; (**B**) Distribution of different MMB-TI classes among germline events (parameters for search: ≥25 bp in length) identified in individual D1 (*n* = 103) (27). (**C**) Similar to (B), but for individual D2 (*n* = 99) (27). (**D**) (i) Complex MMB-TI (CM) event FG-16 found on q11.2 band of Chromosome 3 (red vertical line indicates position; maroon triangles indicate centromere location). (ii) Alignment of event FG-16 consensus to all refence templates. Insertion consists of three parts (orange, red and pink text) copied from all corresponding templates (light blue, purple and green respectively). Microhomology used in each template switching event is underlined. (iii) Schematic illustrating template switching events that lead to the formation of FG-16. Circled numbers indicate the order of template switches. Boxed arrows indicate direction of synthesis, numbers under boxes indicate length of synthesis, and colors correspond to those in (ii). (**E**) Distribution of microhomology analyzed for all resolvable junctions of MMB-TIs from D1 shown in (B) (*n* = 155). Microhomology = identical bases; microhomeology = identical, but with 1bp mismatch or gap allowed. (**F**) Similar to (E), but for D2 shown in (C) (*n* = 148). (**G**) Number of novel MMB-TIs from D1 (from B) and those already existing in NCBI’s Nucleotide collection (nr/nt). (**H**) Similar to (G), but for events from D2 (from C).

We next analyzed the germline variants that were the majority of fibroblast MMBSearch calls identified in the dataset from (27) (Supplementary Data S1). When we set the threshold to signatures of 10 bp or longer, the number of calls was very high in both individuals (between 998 and 1105 calls in individual D1 and between 984–1177 calls for individual D2 (Supplementary Data S1)), and the majority of called events represented microsatellite alterations or other noise. This level of noise was similar to what we observed among the fibroblast-specific calls using these parameters (Supplementary Data S3). To reduce the noise from shorter MMBSearch calls, we narrowed our focus to calls that were at least 25 bp (See Materials and Methods). After filtering out microsatellites and erroneous calls, we found 103 MMBsearch calls from Individual D1 and 99 calls from individual D2 that were further divided into several classes (Figure 2A; Supplementary Data S5, S7). Ten (D1) and 11 (D2) calls were classical MMB-TI events, like those described in (21) (Figure 2A-C (CLSC), and Supplementary Figure S3A, B). Further, 10 (D1) and 14 (D2) insertions were categorized as ‘in-place inversions’ (Figure 2A–C (IPI) and Supplementary Figure S4A), which could either result from MMBIR-like template switching producing an insertion that completely replaces the inverted template, or from a cut-and-paste type non-homologous end joining (NHEJ) mechanism. The finding of microhomologies at the borders of all these in-place inversions supports that these resulted from an MMBIR-like mechanism (Supplementary Figure S5B (IPI); Supplementary Figure S6B (IPI)). Two additional classes of MMB-TI events occurred in the regions of quasi-palindromes. The first were insertions that matched an inverted template within a quasi-palindrome that pre-existed in the reference with a few mismatches, and also matched a second direct orientation template without mismatches (thereby making it the more likely template), which we term, ‘direct duplications’ (Figure 2A (DD); Supplementary Figure S4B). This was the most abundant class, with 42 and 47 direct duplications identified in subjects D1 and D2, respectively (Figure 2B, C). The second class were deletions that resulted from template switching inside the quasi-palindrome, which brought together two pieces of the palindrome that were not previously juxtaposed (Figure 2A (DQP); Supplementary Figure S4C). These events were much less common, with only 8 and 1 event identified in subjects D1 and D2, respectively (Figure 2B, C). Finally, we also observed cases that we termed ‘complex’, because they likely involved several template switching events (Figure 2A (CM) and Supplementary Figure S7). For example, one event (Figure 2D) first copied from an upstream template in the reverse direction, switched to a second template and copied in the forward direction, and then switched to a third template, copying again in the forward direction. This resulted in an insertion originating from 3 distinct templates. We identified 33 complex events in subject D1 and 26 complex events in subject D2 (Figure 2B, C).

We next explored the length of microhomologies mediating the MMB-TI events uncovered by MMBSearch. For all manually verified events, we identified the junctions with resolvable templates and calculated the amount of microhomology present on their borders. We observed that 138 of 155 resolvable junctions from MMB-TI events in D1 and 135 of 148 resolvable junctions from MMB-TIs in D2 contained at least 1 bp of microhomology (Figure 2E, F). In general, all MMB-TI type classes contained junctions with microhomology, though microhomology length varied between types (See Supplementary Data S5–S8, and Supplementary Figures S5 and S6 for microhomology distributions by class). In addition, allowing for a single mismatch or gap to interrupt the complementary bases at junctions revealed additional bases that could have been used as microhomology during the formation of these events. We refer to this interrupted microhomology as ‘microhomeology,’ similarly to the phenomenon described in (6,9,31,32). Because we cannot determine whether these mismatches were present when the event formed, it is impossible to discern whether the event was initiated by annealing at a microhomeologous site or at a site of longer microhomology. In either case, the amount of microhomology that was used to promote the formation of these events could be longer than what is present at the junctions. Together, the presence of microhomologies at the resolvable junctions of the majority of germline events for both individuals indicates that they are most likely the result of template switching driven by microhomology and, therefore, we propose that they result from an MMBIR-like mechanism (Figure 2A), even though other mechanisms cannot be excluded.

Because all MMB-TI events were present in both the blood and in fibroblast clones, we next asked whether they were more likely embryonic or represented common variations of evolutionary origin. However, without the ability to conduct similar analyses on sequencing from the individuals’ parents to differentiate between the two, we used BLAST to instead check whether these insertions matched other known non-reference human genome sequences. We used BLASTn to query NCBI’s nucleotide collection (nr/nt), which includes previously identified non-reference human genomic sequences from diverse populations, to determine whether our MMB-TI events also exist in the genomes of other individuals. We found that only 24 of the 103 events from D1 and 22 of the 99 events from D2 were also found in other studies (Figure 2G, H) and therefore are not likely to be of embryonic origin, as they exist across populations of humans. The majority of the germline events that we identified in both individuals were not found in previously published studies and therefore represent undescribed events that either arose as *de-novo* germline events or represent previously unidentified evolutionary events. In addition, comparison of germline events between the two individuals (D1 and D2), demonstrated that ∼49% (D1) and 51% (D2) of them were common for both individuals, and among them, 30% (D1) and 31% (D2) were those that were not found in NCBI databases (Supplementary Data S5, S7). Because these two individuals are assumed to be unrelated, it is likely that at least these common novel MMB-TI mutations also exist across human populations.

Together, we have identified novel events that were present in human populations, and we propose that both novel and previously identified events were formed through an MMBIR-like mechanism. Interestingly, many of these events represent a broader complexity than what was initially defined by our MMB-TI signature, demonstrating that MMBSearch is capable of identifying a variety of complex events that were not identifiable by other methods.

### Cancer cells accumulate MMB-TI events

**Figure 3.**
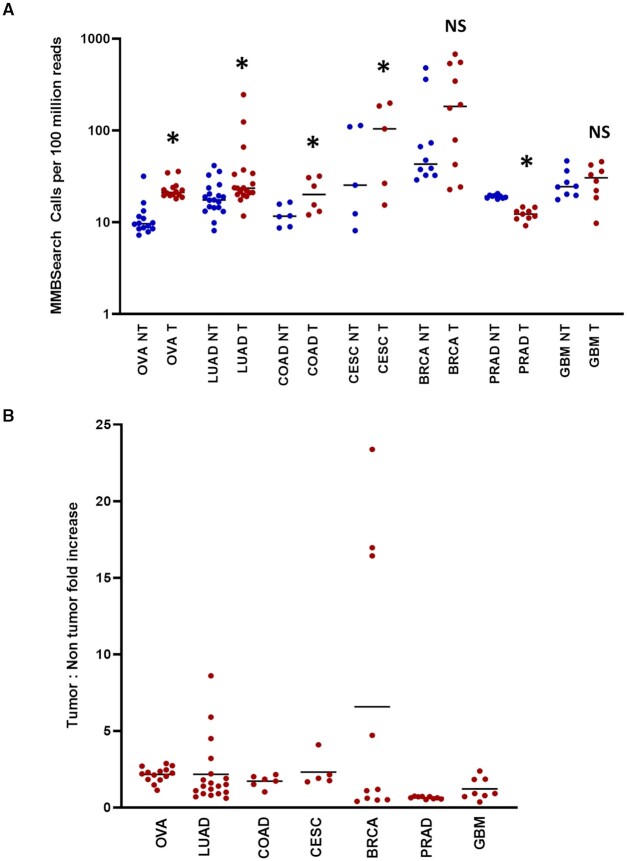
The level of MMB-TIs is increased in tumors from multiple primary tumor sites. (**A**) Number of MMBSearch calls per 100 million reads quantified for paired tumor (red) and non-tumor (blue) samples. WGS data was obtained from 71 total TCGA cancer cases. Asterisks indicate statistically significant difference in the level of MMB-TIs between tumor and non-tumor for each cancer site (*P*-value < 0.05) calculated by ratio paired *t*-test. NS = no significant difference. Cancer primary sites are as follows: Ovarian cancer (OVA) (*n* = 14), lung adenocarcinoma (LUAD) (*n* = 19), colon adenocarcinoma (COAD) (*n* = 6), cervical squamous cell carcinoma and endocervical adenocarcinoma (CESC) (*n* = 5), breast invasive carcinoma (BRCA) (*n* = 10), prostate adenocarcinoma (PRAD) (*n* = 9), and glioblastoma multiforme (GBM) (*n* = 8). Black lines indicate median values for each set. (**B**) Fold-increase in the amount of tumor versus non-tumor MMBSearch calls shown in (A). Each dot represents one cancer case (tumor compared to matched non-tumor). Black lines indicate mean fold-increase.

To determine whether MMB-TIs arise at a high frequency in cancers, we analyzed tumor samples and their matched non-tumor controls obtained from The Cancer Genome Atlas (TCGA) and dbGaP. For this analysis, we extracted all unmapped reads for each sample, analyzed them with MMBSearch using the same parameters for both tumor and matched non-tumor samples, and searched for insertions that were a minimum of 10 bp (See Materials and Methods for details). To accommodate tumor heterogeneity and enable detection of sub-clonal MMB-TI mutations that may exist at very low levels in each sample across varying genome coverages, we set clustering parameters to a minimum of three reads per cluster. We determined the number of MMB-TI events in a total of 71 tumor and matching non-tumor samples obtained from TCGA (Supplementary Data S9). To accommodate varying depths of sequencing coverage across many samples, we normalized the number of MMB-TI events called to the total number of reads for each sample (Supplementary Data S10). Across the seven cancer types analyzed, tumors from ovarian cancer, lung adenocarcinoma, colon adenocarcinoma, and endocervical adenocarcinoma sites had statistically significant increases in the number of calls made by MMBSearch in the tumor samples compared to their matched non-tumor counterparts (Figure 3A; Supplementary Data S10), and average fold increases of 1.7–2.3× (Figure 3B). Breast invasive carcinoma tumors did not show a significant increase overall, but some cases displayed exceptionally high levels of MMB-TIs in the tumor samples compared to non-tumor (4.7- to 23-fold increase). This was also true of some lung adenocarcinoma tumors, which had fold-increases of 3.2- to 8.6-fold (Figure 3B). These data suggest that MMB-TIs arise in tumors from multiple primary sites and accumulate in tumor cells, resulting in elevated levels of MMB-TI events in tumors. We also note that the non-tumor sites in the analyzed patients often contained levels of MMB-TI events that exceed the level that we observed in the fibroblasts of the normal individuals (see Discussion).

**Figure 4.**
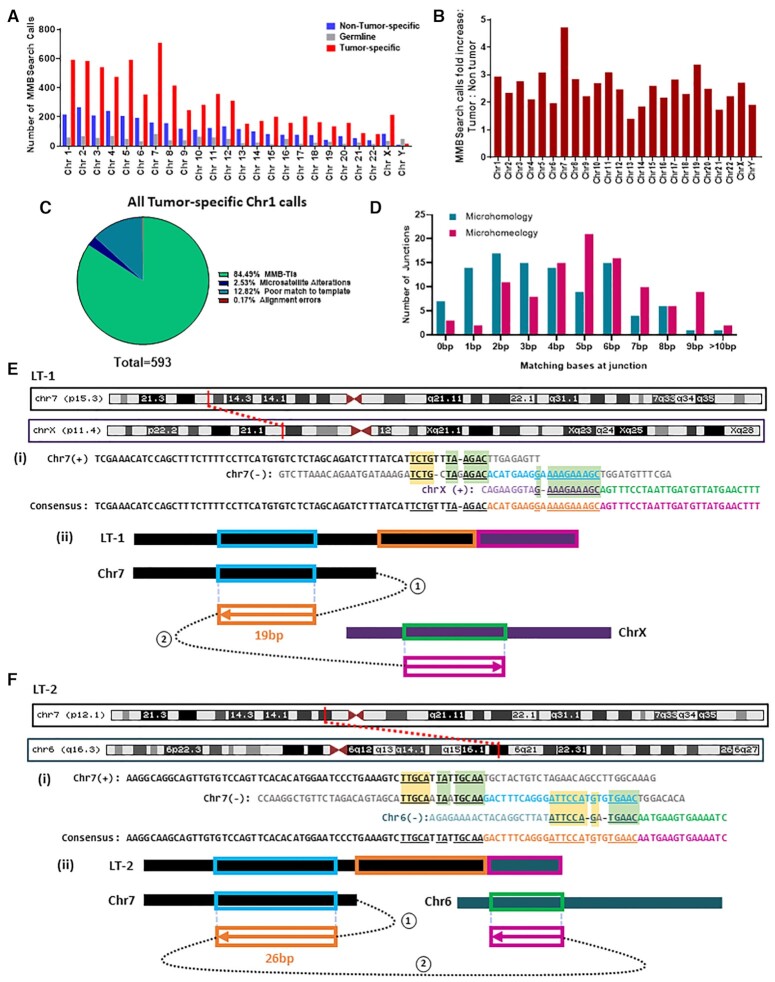
MMB-TIs accumulate in a Lung adenocarcinoma case from dbGaP (accession ID: phs000488.v2.p1). (**A**) MMBSearch calls per chromosome for lung tumor (SRA run ID: SRR556475) and matched non-tumor (SRA run ID: SRR551334) tissue samples. (**B**) Fold-increase of MMBSearch calls in tumor specific-calls compared to non-tumor-specific calls (for the samples shown in A) normalized to the total number of reads for each individual sample. (**C**) Analysis of all MMBSearch calls on Chromosome 1 for lung tumor (*n* = 593). Events were detected using identical parameters to those used in analysis for Figure 3C. (**D**) Microhomology and microhomeology distribution for MMB-TI event junctions from Chromosome 1 (*n* = 103) of the same lung tumor sample. (**E)** MMB-TI event LT-1 found on p15.3 band of Chromosome 7 that is fused with the p11.4 band of Chromosome X. (i) Alignment of event LT-1 consensus to all reference templates. Insertion consists of two parts (orange and pink text) copied from two corresponding templates (light blue and green respectively). Chromosome 7 sequence is shown in black text, and Chromosome X sequence is shown in purple. Microhomology used in each template switching event is underlined and highlighted in light green boxes, and additional microhomology is highlighted in yellow boxes. (ii) Schematic illustrating template switching events that lead to the formation of LT-1. Circled numbers indicate order of template switches, Boxed arrows indicate direction of synthesis, numbers above boxes indicate length of synthesis, and colors correspond to those in (i). (**F**) MMB-TI LT-2 found on p12.1 band of Chromosome 7 that is fused with the q16.3 band of Chromosome 6. (i) Alignment of event LT-2 consensus to all refence templates. Chromosome 7 sequence is shown in black text, and Chromosome 6 sequence is shown in teal. Other text colors and highlights similar to those in (E, i). (ii) Schematic illustrating template switching events that lead to the formation of LT-2. Circled numbers indicate order of template switches, Boxed arrows indicate direction of synthesis, numbers below boxes indicate length of synthesis, and colors correspond to those in (i).

For more detailed analysis and further verification of the MMBSearch results, we chose to focus on lung adenocarcinomas based on our observation that MMB-TI events were significantly elevated in lung adenocarcinoma samples from TCGA, and because it was recently demonstrated that this type of cancer frequently accumulates single-stranded DNA (ssDNA) and also likely uses BIR for DSB repair (33,34), which can enable MMBIR. We chose a stage IV lung adenocarcinoma case from a patient with no smoking history from dbGaP and analyzed the tumor and non-tumor samples with MMBSearch, using identical parameters to those used to analyze all other TCGA datasets. For this patient, after excluding germline MMB-TIs, we observed 2.6-fold more tumor-specific MMB-TI calls compared to matching non-tumor samples (Figure 4A, B; Supplementary Data S11). We next manually inspected all (593) chromosome 1 tumor-specific calls, and found that 84.5% matched the classic MMB-TI (CLSC) pattern (21) (Figure 4C). This high level of MMB-TI events is in stark contrast to the low number of events found on chromosome 1 of one of the fibroblast clones identified in individual D2 when analyzed with the same MMBSearch parameters (Supplementary text; Supplementary Figure S8A; Supplementary Data S12). The remaining 92 (15.5%) calls represented noise, which included alterations of microsatellites and various types of erroneous calls (see Supplementary Figure S1C for descriptions and examples). As an additional control, we isolated genomic DNA from three expanded clonal cultures of lung fibroblast cells and performed WGS using the same library preparation and sequencing method used for the lung tumor samples (see Materials and Methods and Supplementary Text). When analyzed with the MMBSearch tool using identical parameters, we observed that only 3–5% of MMB-TI calls per clone on chromosome 1 matched the MMB-TI signature (CLSC) (Supplementary Figure S8B), which was much lower than the level of observed MMB-TI events found in the lung tumor. We thus conclude that MMB-TIs are highly prevalent in this tumor sample.

Next, to determine the amount of microhomology that mediated the MMB-TI events we identified, we analyzed junctions of 103 MMB-TIs on chromosome 1 by position to determine the length of microhomology used and tolerance for mismatches. We found that 80% of MMB-TI event junctions included microhomology sequences of between 2 and 12 bp (average 3.7 bp), and 95% of junctions contained between 2 and 12 bp of microhomeology, with the average length increasing to 5.1 bp (Figure 4D; Supplementary Data S12 and S13). Therefore, we propose that practically all events were mediated by microhomology or microhomeology, identifying them as authentic MMB-TIs, which were frequent in this cancer sample.

Finally, we also identified several complex MMB-TI events in the tumor sample, which resulted from several consecutive template switching events mediated by microhomologies, and sometimes produced chromosomal fusions (Figure 4E, F; Supplementary Data S14) (see Supplementary text for full description of these events). Notably, in this cancer sample all identified complex and CLSC MMB-TIs had low clonality in the sample (Supplementary Data S14).

### Clonal MMB-TI events lead to disruption of tumor suppressor genes in lung adenocarcinoma

**Figure 5.**
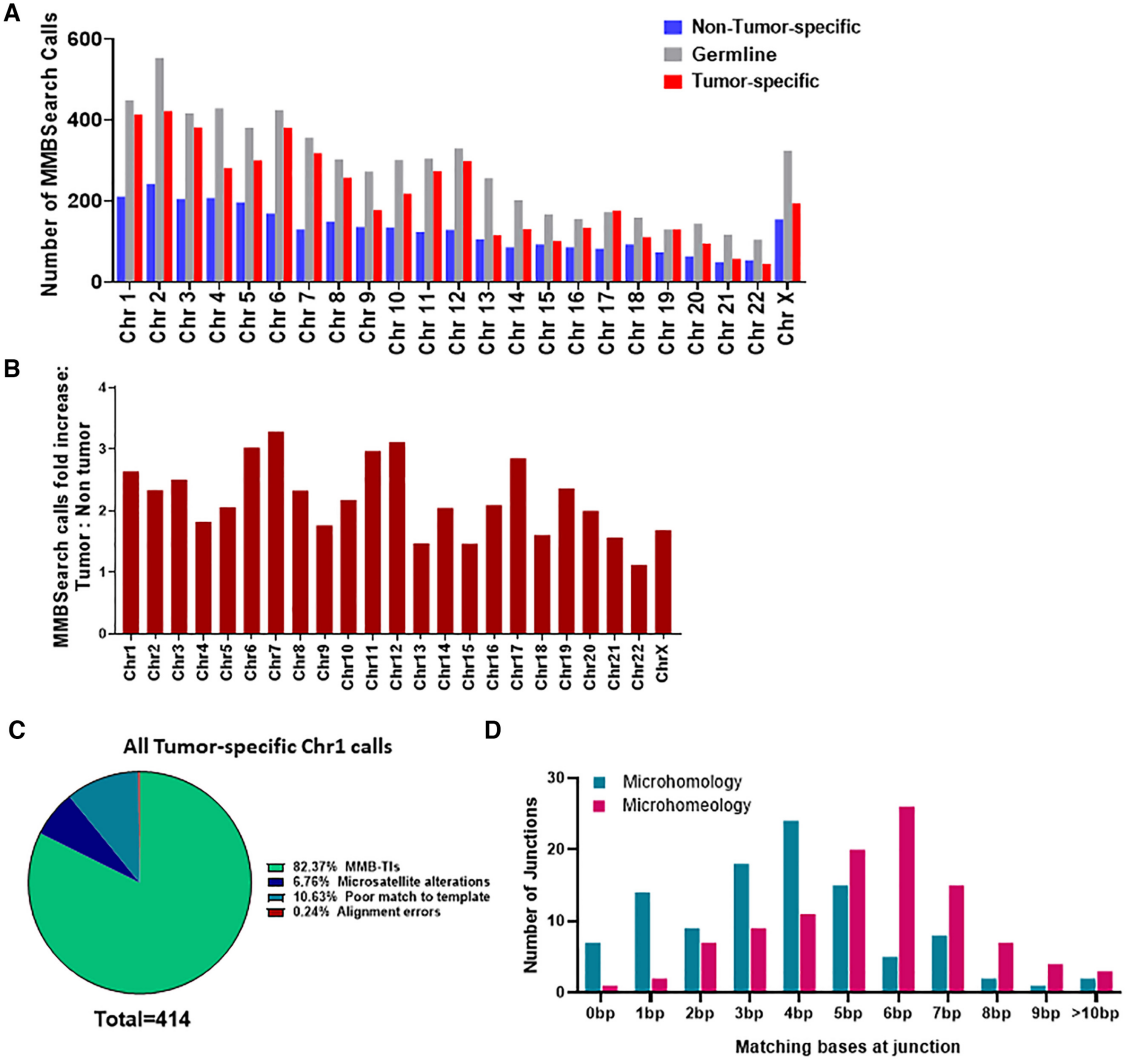
MMB-TI events accumulate *de novo* in a Lung adenocarcinoma patient sample. (**A**) The number of MMBSearch calls per chromosome for lung tumor (BO13-UIBB-1654) and matched non-tumor (BO14-UIBB-1654) tissue samples. (**B**) Fold-increase of MMBSearch calls in tumor-specific calls compared to non-tumor-specific calls (for the counts shown in A) normalized to the total number of reads for each individual sample. (**C**) Analysis of all MMBSearch calls on Chromosome 1 in lung tumor from (A) (*n* = 414). Parameters for both samples were ≥3 reads per cluster, insertions that are ≥10 bp in length, and ≥80% identity between template and insertion. (**D**) Microhomology and microhomeology distribution for lung tumor-specific MMB-TI event junctions (*n* = 105) from Chromosome 1 (dataset shown in A (Chromosome 1) and C).

Due to the limitations of analyzing genomic sequencing data from database sources, namely the inability to confirm particularly complex MMB-TIs by additional methods such as PCR and Sanger sequencing, we next extracted genomic DNA and performed WGS on a stage IB lung adenocarcinoma tumor and matched non-tumor lung tissue samples obtained from a 43-year-old patient with a smoking history. We then analyzed the tumor (BO13-UIBB-1654) and non-tumor (BO14-UIBB-1654) derived reads with MMBSearch using identical parameters to those used to analyze the database-derived cancer samples. After excluding germline calls, we observed that tumor-specific MMB-TI calls were 2.3-fold more abundant compared to non-tumor when normalized to the number of sequencing reads for each sample (Figure 5A, B; Supplementary Data S15 and S16). We manually inspected all (414 total) tumor-specific MMB-TI calls on chromosome 1 (Figure 5C; Supplementary Data S17; Supplementary Figure S1C), and observed that 82% clearly matched the classic MMB-TI (CLSC) pattern. Next, we analyzed junctions of the first 105 MMB-TI events on chromosome 1 by position. We found that 80% of MMB-TI junctions included microhomology sequences of between 2 and 13 bp (average 3.7 bp), and 97% of junctions contained between 2 and 17 bp of microhomeology, with the average length increasing to 5.5 bp (Figure 5D, Supplementary Data S17). Importantly, in this sample junctions with 0, 1 and 2 bp of microhomology ‘acquired’ the most additional bases of microhomeology, averaging 3, 2.9 and 2.6 bp gained, respectively. We thus conclude that MMB-TIs accumulate similarly in this tumor sample to the previously described lung adenocarcinoma sample.

**Figure 6.**
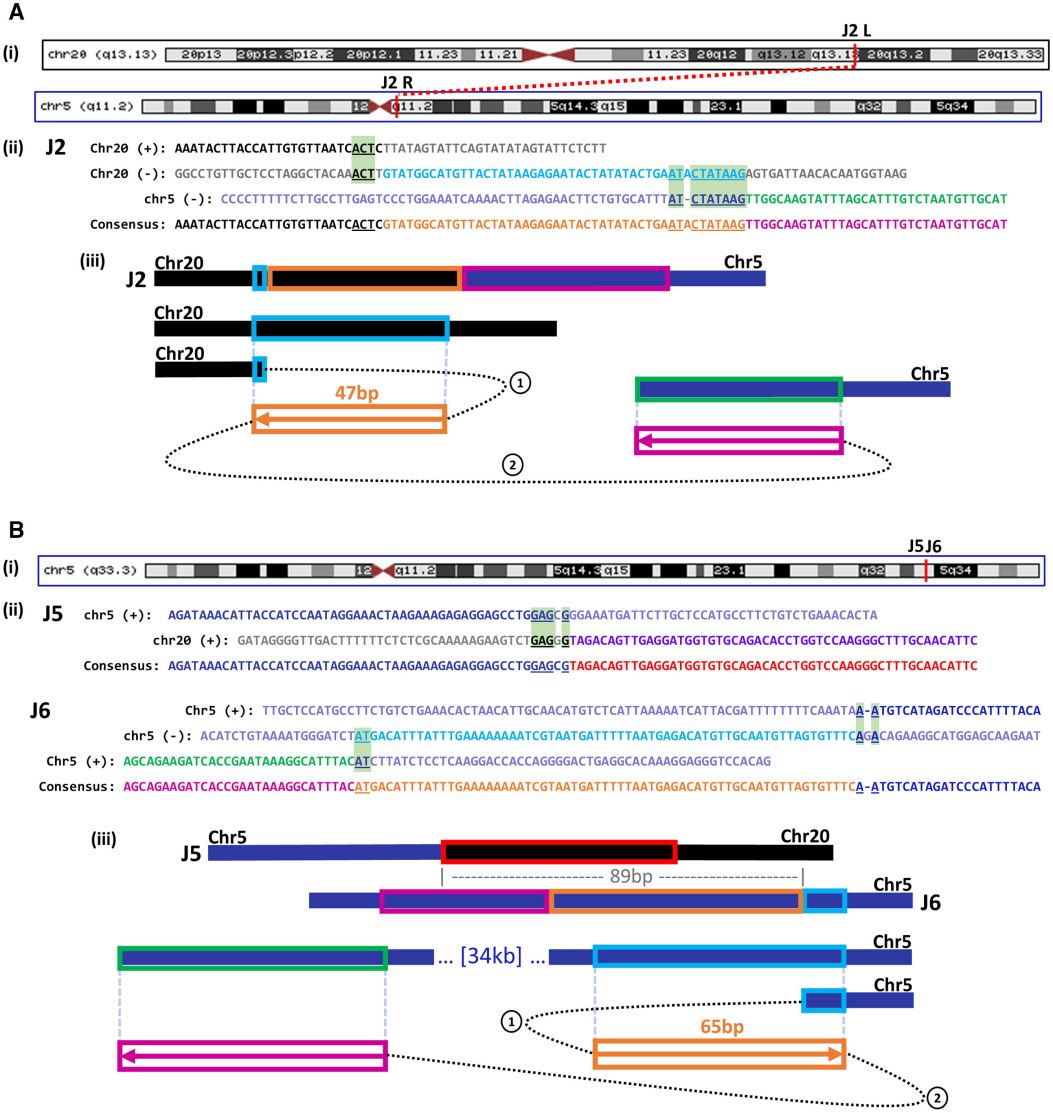
MMB-TI initiates genomic rearrangements. (**A**) (i) MMB-TI event **J2** found on q13.13 band of Chromosome 20 (Chr20) that is fused to the q11.2 band of Chromosome 5 (Chr5) (red vertical lines indicate positions). (ii) Alignment of J2 consensus to all reference templates. Insertion consists of 2 parts (orange and pink text) copied from two corresponding templates (light blue and green, respectively). Chromosome 20 sequence is shown in black text, and Chromosome 5 sequence is shown in navy. Microhomology used in each template switching event is underlined and highlighted in light green boxes. (iii) Schematic illustrating template switching events that lead to the formation of J2. Circled numbers indicate order of template switches. Boxed arrows indicate direction of synthesis, numbers above boxes indicate length of synthesis, and colors correspond to those in (i). (**B**) (i) MMB-TI events **J5** and **J6** found on q33.3 band of Chromosome 5. (ii) Alignment of the J5 rearrangement junction consensus to its reference components on Chromosome 5 and Chromosome 20. Microhomology at J5 is underlined and highlighted in light green boxes. Alignment of event J6 consensus to all reference templates. All labeling similar to (Ai). (iii) Schematic illustrating the structure of J5, its proximity to J6 (89bp), and the two template switching events that lead to the formation of J6. Circled numbers indicate order of template switches. Boxed arrows indicate direction of synthesis, numbers above boxes indicate length of synthesis, and colors correspond to those in **i**.

Though the majority of MMB-TI events detected in this sample were also sub-clonal, we hypothesized that MMB-TIs with higher clonality might represent initial genomic instability in the tumor, or instability that conferred a selective advantage later in tumor development. We identified two events, J2 and J6, that were detected by 14 and 11 reads respectively (Figure 6; Supplementary Data S18). Importantly, in addition to an initial MMB-TI copied from a nearby template, both of these junctions also included a second region copied from a distant template, resulting in chromosomal rearrangements (Figure 6Ai, iii, Bi, iii), which we confirmed by PCR and Sanger sequencing (Figures 6Aii, Bii and 7C; see Supplementary Text for detailed description). The J2 MMB-TI created a fusion between chromosomes 5 and 20 (Figure 6Ai; Supplementary Data S18), likely by initiating on chromosome 20, copying from a nearby template, and switching to chromosome 5 (Figure 6Aiii). Importantly, the junction located on chromosome 20 intersects an intron separating the functional exons of the ADNP gene, making it likely that the resulting protein was affected by the translocation (Supplementary Data S18). Mutations of ADNP, a transcription factor that is part of the SWI/SNF chromatin remodeling complex, have been identified as a cause of neurodevelopmental disorders such as autism (35) and ADNP has been identified as a potential tumor suppressor in breast and colorectal cancers (36,37). Likewise, the J6 MMB-TI created a chromosomal rearrangement that fused two regions of chromosome 5 located 34 kb away from one another (Figure 6Bi; Supplementary Data S18), likely initiating from the region telomere-proximal to the fusion, copying from a nearby template, and switching to the centromere-proximal junction (Figure 6Biii). Similar to the J2 MMB-TI, this event resulted in a fusion between two distinct introns of the EBF1 gene (Supplementary Data S18), which encodes a transcription factor necessary for B-cell progenitor differentiation. Mutations in EBF1 are common in acute lymphoblastic leukemia (38), and heterozygosity of this gene is associated with increased DNA damage and decreased homologous recombination through decreased Rad51 expression and decreased apoptosis in mice (38). Both the J2 and J6 MMB-TIs contained microhomology or microhomeology at their junctions (Figure 6Aii and Bii, see Supplementary text for detailed description).

**Figure 7.**
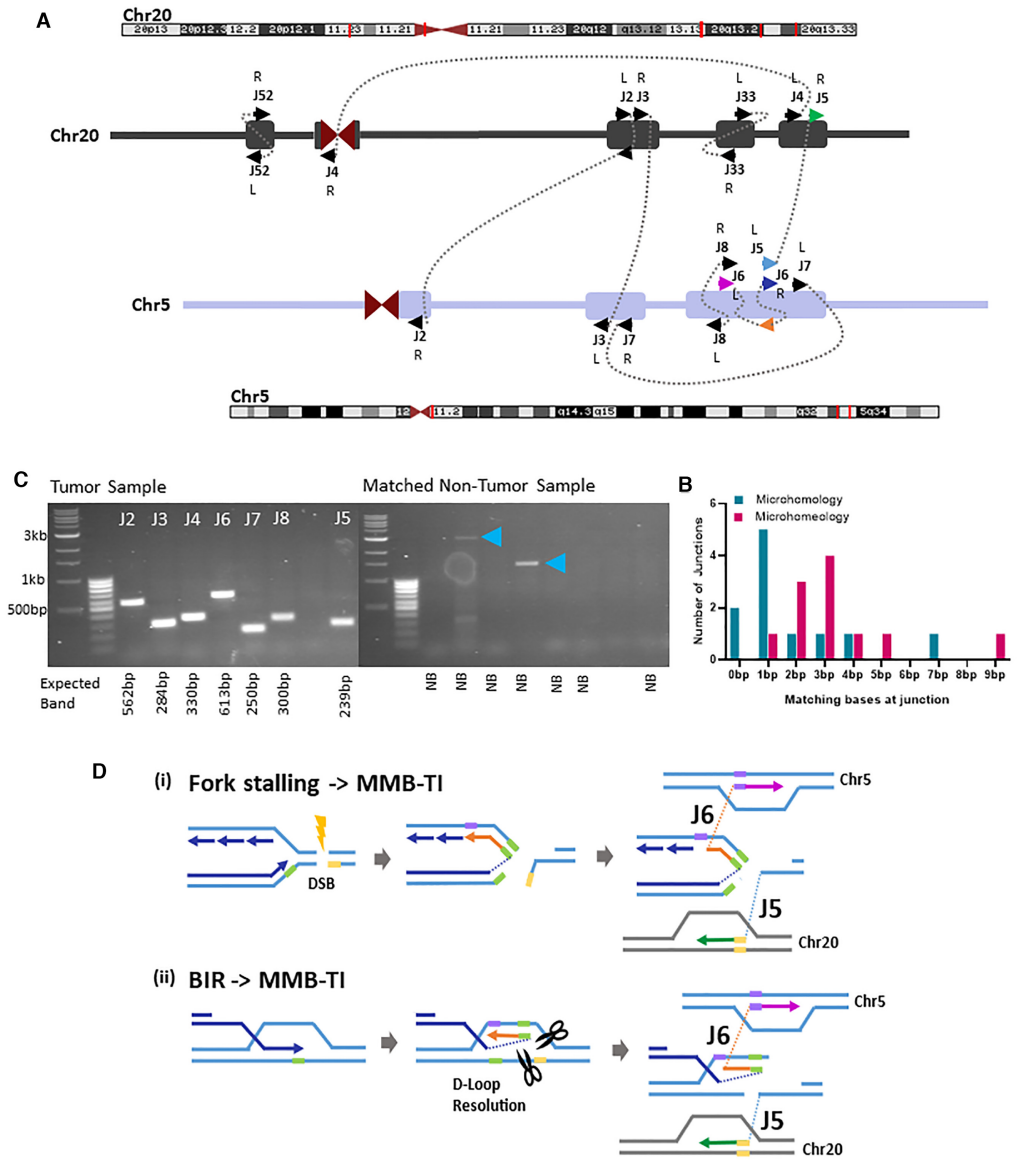
Sub-clonal MMB-TI events associated with multiple additional breakpoints on Chromosomes 5 and 20. (**A**) Schematic of all junctions found on Chromosomes 5 and 20. Chromosome positions (red lines on banding diagram) correspond to each cluster of junctions (Rectangles on schematic). L or R indicates Left or Right side of junction with respect to the 5′ to 3′ reference genome. Arrows points indicate forward (to right) or reverse (to left) strands. Arrow colors correspond to the model shown in (D). (**B**) Distribution of microhomology and microhomeology at all junctions shown in (A) (see Supplementary Data S18 for individual junction counts). (**C**) PCR confirmation of MMB-TI -related rearrangement junctions shown in (A). Primers were designed to anneal at positions 100–300 bp from the rearrangement junctions and expected band sizes are listed below each lane. Blue arrowheads indicate non-specific bands in non-tumor control reactions. (**D**) Two Models for the formation of junctions J5 and J6 shown in (A): (i) replication fork stalling at a damage site (DSB) leads to template switching of the leading strand to the lagging strand template, and subsequent template switching to distant site on Chromosome 5, while a second broken end invades Chromosome 20. (ii) During BIR, the leading strand template switches to the opposite strand within the D-loop structure. D-loop resolution then yields two broken DNA ends that invade different regions on Chromosome 5 and Chromosome 20. Both scenarios lead to genomic rearrangements initiated at an MMB-TI event.

### MMB-TIs initiate genetic instability in a lung adenocarcinoma

The higher read evidence for MMB-TI mutation events J2 and J6, as compared to other MMB-TI mutation calls (Supplementary Data S17), suggests a higher level of clonality of these two mutations in the tumor. Because these events involved chromosomes 5 and 20, we asked whether there were additional junctions nearby on these two chromosomes with similar levels of read evidence that might be related to these MMB-TIs. We found 7 additional breakpoint junctions involving chromosomes 5 and 20 with similar levels of clonality that were confirmed by PCR (Figure 7A, C; Supplementary Data S18 and S19; see Supplementary text for details). These junctions also contained varying lengths of microhomology (Figure 7B; Supplementary Data S18 and S19), and although they did not produce an obvious change in ploidy, they were associated with detectable increases in nearby read counts (Supplementary Figure S9A, B). Analysis of clusters also revealed that the cluster containing MMB-TI event J6 contained a second junction less than 100 bp away called J5 (Figure 6Bii, iii). Based on the proximity of these two independent junctions, it is likely that the J6 MMB-TI initiated template switching that led to discoordination of two ends from a single break, resulting in two distinct rearrangements at a single locus. Because the J6 MMB-TI replaces the majority of its template, we can speculate that it was copied from either the lagging strand in the context of a stalled replication fork (Figure 7Di), or from the top strand within the BIR D-loop (Figure 7Dii), before template switching to a more distal template, while the J5 junction forms as the result of template switching initiated by a second broken end (Figure 7Di, ii). This pattern of coincident junctions was also found in fully clonal rearrangement events (determined by read count), with one MMB-TI identified to be just 63 bp away from a second independent junction (Supplementary Data S18, Junctions J14 and J15; see supplementary text for more details).

We propose that the coincident breakpoints observed at different levels of clonality represent the initial breaks from which all other observed rearrangements with similar levels of clonality proceeded. Further, the presence of MMB-TI events at these coincident junctions implicate MMB-TI as an initiator of genomic rearrangements, which culminate in chromothripsis. Thus, by searching for MMB-TIs with MMBSearch we were able to unravel entire complex rearrangements that are similar to the MMBIR events described in (7,11,17,39) in connection to neurological diseases and chromothripsis.

## DISCUSSION

In this study, we employed our novel MMBSearch software tool to identify MMB-TI events based on the signature of MMB-TI with unprecedented resolution and irrespective of whether they produced a CGR or CNV. This approach allowed us to determine the frequency of MMB-TI events in different cell contexts, which uncovered highly disparate frequencies in normal human cells compared to cancer cells. Specifically, we identified increased levels of MMB-TIs in cancer samples that we sequenced for this study as well as in samples from publicly available databases, where they previously could not be detected. While MMB-TIs were rare in somatic fibroblasts, they were frequent in cancers. Moreover, MMB-TIs were detected only as germline events in fibroblasts, whereas they accumulated over time in cancer cells and contributed to genetic instability. Importantly, our analysis of MMB-TIs identified by MMBSearch allowed us to uncover additional features that were not originally part of our MMB-TI signature. Specifically, the majority of events were associated not only with microhomology, but also with microhomeology, suggesting that it represents a common feature of mammalian MMB-TIs. Also, some of the identified events were much more complex than our original signature, which demonstrated the flexibility of our MMBSearch tool.

**Figure 8.**
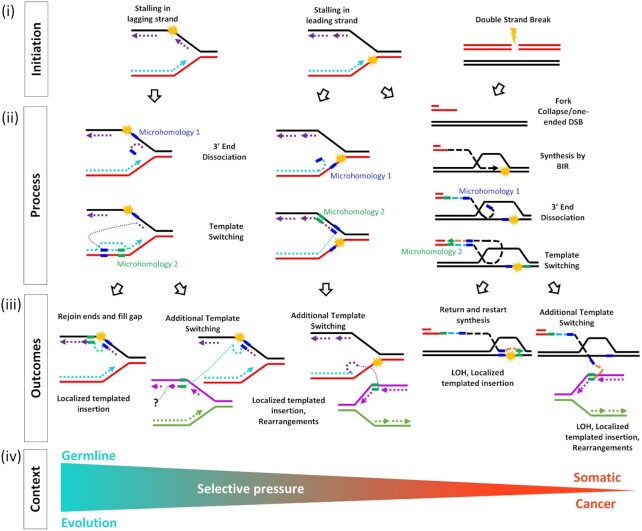
Model explaining formation of MMB-TIs and their possible genomic MMBIR outcomes. (i) Initation: Stalling of leading, lagging strand, or induction of a double strand break lead to replication problems or induction of BIR. (ii) Process: 3′ single-strand (ss) DNA ends are formed by dissociation of a nascent strand from its template during S-phase replication or by 5′ to 3′ resection of DSB ends. Dissociated or resected 3′-ssDNA anneals at microhomology to nearby exposed ssDNA or invades nearby dsDNA at microhomology to prime DNA synthesis. (iii) Outcomes: Additional rounds of template switching, or invasion mediated by microhomology can lead to either a return to the original template or more complex rearrangements. (iv) Context: MMB-TI events that do not disrupt genes are more likely to accumulate as germline variants over the course of evolution. More complex MMB-TI events, especially those that disrupt genes or lead to gross chromosomal rearrangements, may only appear in pathogenic contexts.

Based on our data, we propose a model explaining the formation of MMB-TIs that can lead to MMBIR outcomes (Figure 8). In this model, as a result of fork stalling or induction of a double strand break (Figure 8i), a 3′ single-strand DNA (ssDNA) end formed by dissociation of a nascent strand from its template during S-phase replication, or by 5′-to-3′ resection of DSB ends, can anneal to exposed ssDNA at a site of microhomology, or invade double-strand DNA (dsDNA) using microhomology to prime synthesis (Figure 8ii). Subsequent template switching events can either return to the original template or anneal elsewhere, resulting in a more complex rearrangement (Figure 8iii). More localized MMBIR events that do not disrupt genes may accumulate as germline variants over the course of evolution, while more complex MMBIR events, especially those that disrupt genes or lead to CGRs, are more likely to appear only in pathogenic contexts (Figure 8iv). According to our model, MMBIR can result from interruption of BIR or (directly) from the interruption of S-phase replication. In both cases, the MMB-TIs could represent the initiating step of MMBIR.

### Initiation of MMB-TI often involves microhomeology

Our analysis determined that template switching leading to MMB-TIs involved microhomologies, which is consistent with our proposed molecular mechanism (Figure 8). Previous attempts to determine the microhomology requirements for chromothripsis (17) suffered from collectively analyzed events originating from different repair mechanisms, making it impossible to determine the microhomology requirements for MMBIR specifically. Our analysis of MMB-TI events (often leading to MMBIR) in lung adenocarcinomas demonstrated that nearly all MMB-TI events involved a significant amount of microhomology. We further report that these microhomologies can often be extended by allowing gaps or mismatches. In particular, we observed that addition of tolerance for a single unmatched base (gap or mismatch) universally extended microhomology at all junctions, but especially at those that were previously recorded as having 0 to 1 bp of microhomology. The implications of this are two-fold. First, it suggests that the actual amount of homology typically used for MMB-TI (and likely for all cases of MMBIR) is higher than previously appreciated. Second, it indicates that the annealing (pairing) between strands initiating the template switching that produces MMB-TIs does not have to be perfect. In fact, based on our findings, even the 3′-most base does not have to be matched to the template to prime synthesis.

The concept of imperfect microhomology, or microhomeology, that we used in this work is different from what has been described in previous studies that attempted to account for microhomology containing mismatches (31). The authors attempted to define microhomeology by gapped alignment of junction-flanking sequences, which demonstrated that microhomology could be imperfect, but did not specify cases with a mismatch at the 3′ end implicated in priming the synthesis. In our analysis, in which we identified unmatched bases beginning from the 3′ end, we were able to demonstrate that MMB-TI can be initiated even when the bases closest to the 3′ end are mismatched. The observation that MMB-TI can utilize microhomeologous sequences allows us to speculate that formation of MMB-TI is unlikely to be carried out by polymerase(s) that conduct S-phase DNA synthesis, which possess efficient exonuclease function that would be expected to remove mismatched bases before extending. Translesion polymerases are likely candidates for this function. Indeed, in yeast, we previously demonstrated that polymerase ζ is responsible for MMB-TI formation. (21).

Another candidate polymerase to initiate synthesis from imperfect primers in mammalian cells is Polθ, which is known to form insertions and deletions by initiating DNA synthesis from primers annealed at microhomologies in theta-mediated end-joining (TMEJ) (40,41). It has been recently shown that, during DSB repair, TMEJ favors annealing of a broken 3′ end at microhomologies most proximal to DSBs (42). Furthermore, Polθ has been shown to aid in repair of collapsed replication forks (43) which can serve as substrates for the initiation of MMBIR. It is possible that Polθ-mediated TMEJ may represent at least a subset of the MMB-TI events that we uncovered in this study. Because the literature regarding TMEJ has focused on shorter, less complex insertions than we associate here with MMB-TIs, we propose that the events we describe here as MMB-TIs are likely distinct from those previously described as TMEJ.

### MMBSearch as a tool to detect MMB-TIs

In this study, we used our new software tool to detect the initial, and sometimes multiple template switches of MMB-TI events. Consistent with our initial hypothesis, many of these events were discarded by conventional BWA mapping methods, where they were either unmapped or among clipped reads, depending on the parameters of mapping. We previously developed a software tool for direct detection of MMBIR (MMB-TI) events in the yeast genome (44), but that approach lacked the efficiency and flexibility required to query larger and more complex datasets of human cancer genomes for discovery of potentially longer and more complex MMB-TI events. While our data here show that MMB-TIs accumulate massively in cancer, the previous tool discovered very few MMB-TIs when piloted on a breast cancer genome, suggesting poor sensitivity when analyzing human cancer genomes. In creating MMBSearch, we re-designed the core of the previous tool to increase sensitivity and to better inform the search algorithm with what is known about MMB-TI/MMBIR in human genomes from studies of human diseases (8,10,11,16,17,19,20). Specifically, we implemented a new clustering algorithm that allows for the accurate discovery of MMB-TIs that shift sequence reading frame that were thrown out by the previous tool, we added a novel parallel computing approach that makes MMBSearch suitable for identifying MMB-TI signatures far more efficiently within complex datasets like cancer genomes that exhibit massive genome instability, and we added additional flexibility to parameters that allow MMBSearch to call longer, multi-templated, and non-returning MMB-TI events. Based on our data here, MMBSearch accurately calls MMB-TIs in greater quantities and with greater complexity than its predecessor. Its sensitivity is especially high for identifying insertions without replacement, and somewhat lower for insertions with replacement, which likely results from the leveraging of reads that differ greatly from the reference. Furthermore, by adding more flexible parameters for the comparison of MMB-TIs to their templates, MMBSearch can discover complex multi-template MMB-TI events. In the future, one possible improvement could be to increase the search distance for MMB-TI templates from very nearby (100 bp) to genome-wide. This would allow us to identify all MMB-TI /MMBIR events, including those that have templates further away, potentially on other chromosomes.

The results from the present study indicate that MMBSearch should be automated in the future to enable identification of subsequent template switches to distant and nearby templates. This currently requires manual curation of events to identify additional templates for highly complex MMB-TI events. Automation of this process would contribute to our understanding of the mechanism that generates complex MMB-TI/MMBIR patterns like those discovered in this study. In addition, automation for determining the amount of microhomology and microhomeology associated with MMB-TI represents another important goal.

### MMB-TI: a driver of cancer evolution?

Our results suggest that normal human cells differ from cancer cells in their propensity for MMB-TI. In the genomes of normal skin fibroblasts from two individuals, MMB-TI signatures were only present as germline variants, which differentiates MMB-TI events from all other mutations, including base substitutions and frameshifts that have been observed to accumulate with age in these cells (27). Some of the MMB-TIs were also found as a subset of germline events across human populations from other studies, or were shared between the two individuals, implying that these events may have accumulated over the course of human evolution. While these germline MMB-TIs could be complex, having copied from up to five templates to form a single event, they generally did not lead to CGRs. This is expected, as the selective pressure imposed during evolution would likely eliminate detrimental MMB-TIs from the genome, except in some rare cases of congenital diseases.

In tumors, across a variety of TCGA cohorts, our results indicate that MMB-TIs accumulate *de novo*. Most accumulated MMB-TIs were sub-clonal, but in our detailed analysis of lung adenocarcinomas, we specifically found some rare MMB-TIs tied to GCR junctions that had expanded in the tumor. Importantly, we observed these expanded MMB-TI events disrupting DNA repair (EBF1) and chromatin remodeling (ADNP) genes. Based on this observation, we propose that MMB-TIs may play a critical role in cancer initiation, progression, and innate drug resistance by mutating tumor suppressor or oncogenes. For tumors treated with chemo- or radiotherapy, in which DSBs are induced *en masse*, MMB-TIs may play a key role in adaptive response and disease relapse. A more comprehensive understanding of how the mutational landscape of tumor cells influences the propensity for MMB-TIs, and likely for MMBIR is needed to determine why some rearrangements may be preferentially selected in certain genetic contexts.

In donors of tumors where MMB-TI events accumulated, we often found high levels of MMB-TIs in matched normal tissues as well. Although there was a general trend of increased MMB-TI load in tumor compared to matched normal samples, the relatively high MMB-TI load in normal tissue suggests that MMB-TI events may still accumulate in some tissues of cancer patients, even though our analysis of clonal fibroblast lineages of healthy individuals did not uncover accumulation of MMB-TIs. This may have been influenced by the mutational landscape of the donors of fibroblast versus donors of tumor tissue, or it may indicate that the predisposition of different tissue types to undergo DNA damage and repair can influence the frequency of MMB-TIs. These questions provide a blueprint for many future studies that will require a wide breadth of new analyses to answer, yet our work here provides the first proof of principle aimed to demonstrate that thousands of MMB-TIs can be found in cancer genomes and that different tissues and/or individuals may vary in their numbers. In the future, extensive analysis of DNA samples from different tissues and across populations of healthy donors would be required to improve our understanding of how and where MMB-TIs accumulate. One other possible source that could contribute to the finding of elevated MMB-TI events in non-tumor samples is contamination by tumor DNA, particularly if tumor cells are actively being destroyed by treatment or the immune system. Studies have found tumor DNA circulating in the blood as cancer cells are eliminated (45–47), and identification of tumor-specific mutations from circulating tumor DNA (ctDNA) in the peripheral blood is the basis for cancer diagnostic blood tests (48,49). This provides one explanation for why some matched non-tumor samples, particularly whole blood, have a high frequency of MMB-TIs, and suggests that future studies to measure MMB-TI signature load in blood could be useful as a diagnostic or prognostic biomarker for some types of cancer.

## DATA AVAILABILITY

The MMBSearch tool package and all additional code associated with this study, including the code used to generate artificial datasets, can be downloaded from https://github.com/malkovalab/MMBSearch. The majority of the datasets analyzed in this study are available from the The Cancer Genome Atlas (TCGA) project (https://portal.gdc.cancer.gov/) or the database of genotypes and phenotypes (dbGaP) authorized access portal (https://www.ncbi.nlm.nih.gov/gap/). Accession IDs for all samples from databases can be found in Supplementary Data S2, S3, S9 and S11 as either SRA run IDs or GDC case IDs. Raw sequencing reads from all prepared genomic DNA are available through dbGaP (study accession phs002237.v1.p1). All data supporting the findings of this study are represented in this article or supplementary materials. Raw program output files are available upon reasonable request.

## Supplementary Material

gkab685_Supplemental_Files

## Notes

Present address: Beth Osia, Department of Cancer Genetics and Epigenetics, Beckman Research Institute, City of Hope, CA 91010, USA.

Present address: Tyler Jackson, Department of Cancer Genetics and Cell Biology, Baylor College of Medicine, Houston, TX 77030, USA.
