# Supplementary material for: Cancer cells are highly susceptible to accumulation of templated insertions linked to MMBIR
Source: Nucleic Acids Res. 2021 Aug 11;49(15):8714–31. doi: 10.1093/nar/gkab685 (PMC8421209; doi:10.1093/nar/gkab685)
Supplement: gkab685_Supplemental_Files [file gkab685_supplemental_files.zip › Osia et al 2020 Supplemental corrected AM 0725.pdf]

Supplementary Information

Cancer cells are highly susceptible to accumulation of templated insertions linked to MMBIR

Beth Osia[1], Thamer Alsulaiman[2], Tyler Jackson[1], Juraj Kramara[1], Suely Oliveira[2], and Anna Malkova[1*]

Supplementary Figure Legends

Supplementary Figure 1: Overview of analysis methods and manual categorization of MMBSearch results.

A MMBSearch Parameters used to analyze fibroblast and cancer datasets. Different parameters used for quantitative analysis (MMBSearch call counts), and detailed analysis (categorization and manual curation of MMB-TI calls and breakpoints)

B Overview of methodology for comparison of MMBSearch calls between paired samples (e.g. blood and fibroblast lineage samples or non-tumor and tumor samples). MMBSearch candidate clusters are resolved to consensus reads (See Figure 1B for details). For consensus reads where MMB-TI events are found, the position of each MMB-TI is compared to the positions of all consensus reads of the paired sample. MMB-TI events from one sample that intersect a consensus read of the other sample are considered to be common between the samples or germline, while MMB-TIs that do not intersect are considered to be sample-specific.

C Descriptions and examples of how MMBSearch results are manually categorized. Examples of non-MMB-TIs (Microsatellite alterations and Excluded call types) are shown on the right. Asterisks indicate matching bases at the bottom of the alignments shown. Abbreviations: "Ref" = Reference sequence. MSA= Microsatellite alteration; , AE=Alignment error; SEC= sequencer error contamination-calls due to poly-A runs or adapter contamination at the ends of reads; UNK= unknown; reported insertion contained many mismatches to the template, was shorter than the required length (10bp), or was otherwise undeterminable.

Supplementary Figure 2: MMBsearch calls for all fibroblast clones.

MMBSearch calls (from clusters with ≥10 reads, insertions that are ≥10bp in length, and ≥80% identity between template and insertion) per chromosome for 10 fibroblast cell lineages from the study of (1). Germline calls are those found to overlap by position between both the blood and fibroblast clones.

A Individual 1: left hip (D1-L-H), right hip (D1-R-H1 and D1-R-H2), left forearm (D1-L-F1 and D1-L-F2), and right forearm (D1-R-F).

B Individual 2: left hip (D2-L-H), right hip (D2-R-H), left forearm (D2-L-F), and right forearm (D2-R-F).

Supplementary Figure 3: Examples of Classic MMB-TI events found in germline of fibroblast samples.

A Classic MMB-TI (CLSC) FG-143 found on q11.21 band of Chromosome 7 of individual D1. **(i)** Read cluster output generated by MMBSearch for FG-143. Cluster contains a 39bp insertion (orange box). **(ii)** Alignment of event FG-143 consensus to reference template. The insertion is shown in orange text and the template in light blue. Microhomology used in each template switching event is underlined. **(iii)** Schematic illustrating template switching events that lead to the formation of FG-143. Circled numbers indicate the order of template switches. Boxed arrows indicate the direction of synthesis, numbers under boxes indicate the length of synthesis and colors correspond to those in **(ii)**.

B CLSC event FG-20 found on q25.32 band of Chromosome 3 of individual D2. **(i)** Read cluster containing an 85bp insertion (orange box). **(ii)** Alignment of event FG-20 consensus to reference template. Insertion is shown in orange text with its template (light blue). Microhomology is underlined. **(iii)** Schematic illustrating template switching events that lead to the formation of FG-20. Circled numbers indicate the order of template switches. Boxed arrows indicate direction of synthesis, numbers under boxes indicate length of synthesis, and colors correspond to those in **(ii)**.

Supplementary Figure 4: Examples of several classes of MMB-TI events found in germline of fibroblast samples.

A Representative In-place Inversion (IPI) event FG-106 found on p21 band of Chromosome 2 of individual D1. **(i)** Alignment of event FG-106 consensus to reference template. Insertion (orange text) is 27bp and completely replaces the template (light blue text) end-to-end. Microhomology is underlined. **(ii)** Schematic illustrating one possible configuration of template switches. Circled number indicates the order of template switches. Boxed arrow indicates direction of synthesis, numbers under boxes indicate length of synthesis, and colors correspond

to those in **(i)**.
B Representative Direct Duplication (DD) event FG-129 found on q22.1 band of Chromosome 5 of individual D1. **(i)** Alignment of event FG-129 consensus to best reference template. Insertion (orange text) is 42bp and best aligns to a forward template (also in orange text). The inverted template found by MMBSearch is highlighted in light blue. Microhomology is underlined. **(ii)** Schematic illustrating one possible configuration of template switches. Circled number indicates the single template switch. Boxed arrow indicates direction of synthesis, numbers under boxes indicate length of synthesis, and colors correspond to those in **(i)**, including the light blue box showing the location of the inverted template.
C Representative Deletion at a Quasi-palindrome (DQP) event FG-173 found on q24.23 band of Chromosome 12 of individual D1. (i) Alignment of event FG-173 consensus to reference deletion junction locations. Palindromic sequences are highlighted in purple. Microhomology is underlined. **(ii)** Schematic illustrating the template switching event that resulted in a 224bp deletion between two 76bp inverted repeats (purple arrows). After the deletion, 22bp of the left repeat, and 35bp of the right repeat remained. Circled number indicates the single template switch. Colors correspond to those in **(i)**.

Supplementary Figure 5: Microhomology distributions for all MMB-TI classes found in germline of fibroblast clones of individual D1.
A Microhomology (Green bars) and Microhomeology (Pink bars) distributions for simulated MMB-TI events (Expected) and all classes of MMB-TIs found in germline of fibroblast clone SRR4047717 (Observed). The "Expected" microhomology and microhomeology probability distribution is based on sampling of artificial randomly inserted MMB-TIs on Chromosome 17 (data set used in Figure 1D) and multiplying by the total 155. D1 MMB-TI junctions analyzed in "Observed".
B Distributions of microhomology and microhomeology for different classes of MMB-TIs (CLSC, CM, IPI, DD, and DQP (see Figure 2A for abbreviations)) that make up all classes shown in **A** (Observed) from individual D1 (See Supplementary Data S6 for details).

Supplementary Figure 6: Microhomology distributions for all MMB-TI classes found in germline of fibroblast clones of individual D2.
A Microhomology (Green bars) and Microhomeology (Pink bars) distributions for simulated MMB-TI events (Expected) and all classes of MMB-TIs found in germline of fibroblast clone SRR4047718 (Observed). The "Expected" microhomology and microhomeology probability distribution is based on sampling of artificial randomly inserted MMB-TIs on Chromosome 17 (data set used in Figure 1D) and multiplying by the total 148. D1 MMB-TI junctions analyzed in "Observed".
B Distributions of microhomology and microhomeology for different classes of MMB-TIs (CLSC, CM, IPI, DD, and DQP (see Figure 2A for abbreviations)) that make up all classes shown in **A** (Observed) from individual D1 (See Supplementary Data S4 for details).

Supplementary Figure 7: Examples of Complex MMB-TI events found in germline of fibroblast samples.
A Complex MMB-TI (CM) event FG-16 found on q11.2 band of Chromosome 3 of individual D2. **(i)** Read cluster output created by MMBSearch for FG-16. Read cluster contains a 45bp insertion which consists of 3 parts, each copied from different templates (distinguished by 3 colored boxes). **(ii)** Alignment of event FG-16 consensus to all refence templates. Insertion consists of 3 parts (orange, red, and pink text) copied from all corresponding templates (light blue, purple, and green respectively). Microhomology used in each template switching event is underlined. **(iii)** Schematic illustrating template switching events that lead to the formation of FG-16. Circled numbers indicate the order of template switches. Boxed arrows indicate direction of synthesis, numbers under boxes indicate length of synthesis, and colors correspond to those in **(ii)**.
B CM event FG-60 found on q21.13 band of Chromosome 9 of individual D2. **(i)** Read cluster output generated

by MMBSearch for FG-60. Read cluster contains a 73bp insertion which consists of 3 parts, each copied from different templates (distinguished by 3 colored boxes). **(ii)** Alignment of event FG-60 consensus to all reference templates. Insertion consists of 3 parts (orange, red, and pink text) copied from all corresponding templates (light blue, purple, and green respectively). Microhomology used in each template switching event is underlined. **(iii)** Schematic illustrating template switching events that lead to the formation of FG-60. Circled numbers indicate the order of template switches. Boxed arrows indicate direction of synthesis, numbers under boxes indicate length of synthesis, and colors correspond to those in **(ii)**.

Supplementary Figure 8: Comparison of MMBSearch calls from Chromosome 1 of lung tumors, and fibroblast and cultured lung fibroblast controls.

A Analysis of Chromosome 1 MMBSearch calls (from clusters with ≥3 reads, insertions that are ≥10bp in length, and ≥80% identity between template and insertion) for IV Lung tumor (BO13-UIBB-1654), IB Lung tumor (SRR556475) obtained from dbGaP, and Fibroblast D2-L-F (SRR4047705) from (1). Germline MMBSearch calls were excluded for these sets. "MMB-TIs" are those that resemble the Classic MMB-TI pattern (CLSC).

B Analysis of Chromosome 1 MMBSearch calls (parameters identical to **A**) for 3 Lung fibroblast clones BO8, BO2, and BO4. All MMBSearch calls were analyzed without exclusion of common calls. "MMB-TIs" are those that resemble the Classic MMB-TI pattern (CLSC).

Supplementary Figure 9: **Read aggregation maps show copy number increases on Chromosomes 5 and 20 in IB Lung tumor sample.**

A Read aggregation tracks for entire Chromosomes 5 and 20 of tumor and non-tumor samples from IB Lung tumor. Locations of MMB-TI junctions J2 (R: right side, L: left side) and J6 are indicated with red arrowheads. Read aggregation levels for different ploidies are indicated with red dotted lines.

B Zoomed-in read aggregation tracks for the locations of J6 and J2 (R: right side, L: left side) in tumor sample, indicated as red lines.

Supplementary Data.

Supplementary data S1. Summary of MMBSearch calls from 10 clonal fibroblast lineages of 2 individuals.

Supplementary data S2. MMBSearch analysis of fibroblasts of individual D1 from (1).

Supplementary data S3. MMBSearch analysis of fibroblasts of individual D2 from (1).

Supplementary data S4. MMBSearch output for fibroblast-specific MMB-TI call with analysis.

Supplementary data S5. Analysis of germline events and microhomology in individual D2 from SRR4047718 (D2-R-F) from (1).

Supplementary data S6. Maps of all germline MMB-TI events found in sample SRR4047718 (D2-R-F) from (1).

Supplementary data S7. Analysis of germline events and microhomology in individual D1 from SRR4047717 (D1-L-F1) from (1).

Supplementary data S8. Maps of all germline MMB-TI events found in sample SRR4047717 (D1-L-F1) from (1).

Supplementary data S9. List of all TCGA samples analyzed by MMBSearch.

Supplementary data S10. Summary of analysis of sample-specific MMBSearch calls from TCGA cancer genomes.

Supplementary data S11. Summary of sequencing and MMBSearch Calls for tumor (SRR556475) and matched non-tumor (SRR551334) samples from stage IV lung adenocarcinoma patient from dbGaP.

Supplementary data S12. Analysis of all tumor-specific MMBSearch calls on chromosome 1 from sample SRR556475.

Supplementary data S13. MMBSearch output for all chromosome 1 tumor-specific results for sample SRR556475.

Supplementary data S14. Tumor-specific complex MMB-TI junctions from sample SRR556475.

Supplementary data S15. MMBSearch output for all chromosome 1 tumor-specific results for sample BO13-UIBB-1654.

Supplementary data S16. Summary of sequencing and MMBSearch calls for tumor (BO13-UIBB-1654) and matched non-tumor (BO14-UIBB-1654) samples from stage IB lung adenocarcinoma patient.

Supplementary data S17. Analysis of all tumor-specific MMBSearch calls on chromosome 1 from sample BO13-UIBB-1654.

Supplementary data S18. Clonal and sub-clonal tumor-specific MMB-TI and SV junctions from sample BO13-UIBB-1654.

Supplementary data S19. Maps of breakpoint junctions listed in supplementary data S18.

Supplementary data S20. Primers for confirming Tumor-specific (BO13-UIBB-1654) junctions of selected MMB-TIs and SVs by PCR and Sanger sequencing.

Supplementary text

Testing MMBSearch sensitivity with artificially generated read sets

To test the sensitivity of the MMBSearch in finding the MMB-TI pattern, we created a set of synthetic genomes that each contained 993 MMB-TI insertions 20-50 bp in size on human Chr 17 (See Materials and Methods for details). From these synthetic genomes, we generated 125bp paired-end artificial Illumina sequencing reads with an average insert size of 500bp and amounting to 30x average coverage across chromosome 17 using the NGS read simulator ART (2). After analyzing these read sets with the MMBSearch, recall (sensitivity) and false positives were calculated for each length and type of insertion by comparing the MMB-TI insertions called by the program to the list of insertion sequences within a 10% tolerance (e.g. 5bp for a 50bp insertion). Recall was highest for longer insertions that did not replace sequence (92-97% for insertions without replacement, and 65-77% with replacement) (Figure 1C, D), which is likely because the MMBSearch leverages reads that differ greatly from the reference and relies on local alignment to accurately differentiate between the insertion and its flanking reference-matching sequence, making more disruptive insertions easiest to identify and more likely to have their insertions called in full. Likewise, shorter insertions and those that replace sequence are more prone to partial calls. The number of false positives (see Materials and Methods) detected by the program was between 8 and 34 for various insertion lengths, representing 1-3% of calls made by MMBSearch. These false positives are caused solely by misalignment of half-reads to some repetitive regions of chromosome 17.

MMB-TI events do not accumulate with age in human fibroblasts.

To test our hypothesis that MMB-TI events accumulate with age like other types of mutations identified by (1), we analyzed sequencing reads from all 10 clonal fibroblast cell lineages and their matched blood samples (6 clones from individual D1 in the study and 4 clones from individual D2). Because the fibroblast lineages were clonal, we required a minimum of 10 reads per cluster to exclude any MMB-TI that may have arisen during clone expansion (see Materials and Methods for details). We set parameters for MMBSearch to call insertions with a minimum length of 10 bp and an identity threshold of 80% between the insertion and its template. MMB-TI events called at the same reference positions in both fibroblasts and blood were considered germline variants

(present from the zygotic stage), while those that were found only in fibroblast clones were considered fibroblast specific. Strikingly, we observed that the majority (74-93%) of all MMBSearch calls among all 10 fibroblast clones were germline variants (Supplementary Figure 2; Supplementary Data S1, S2, S3). The remaining 7-26% of MMB-TI calls we identified as fibroblast-specific events; however, all but one call was either present among multiple fibroblast clones, represented microsatellite alterations, or were determined to be false-positive outcomes (Supplementary data S1, S2, S3). The sole remaining fibroblast-specific variant was a 354-kb deletion on Chr3 in the first left forearm sample of Individual D1 (D1-L-F1) previously described in (1) (Supplementary Data S4). This deletion occurred at a short 5 bp quasi-palindrome (See Figure 2A - DQP), with just 1 bp of microhomology at the junctions. However, this event appears to differ from similar germline events (Supplementary Figure 4C; Supplementary Data S5-S8) in both size of the deletion and amount of microhomology. Therefore, it is unlikely that this event represented MMB-TI. Taken together, we found no evidence of MMB-TI accumulation in fibroblasts with age, which represents an intriguing difference between MMB-TI mutational events and other mutations that accumulated at high frequency in aging fibroblasts (1).

Description of sub-clonal complex MMB-TI events in lung cancer from dbGaP

By surveying Chromosomes 1-7, we found 41 sub-clonal events (defined as being present in fewer reads than 30% of the average read coverage for the sample) that, in addition to an insertion copied from a nearby template, contained sequence copied from a separate template (Supplementary Data S14), making them similar in structure to the events shown in Figure 4. For these complex events, 14 of the secondary templates were identified using BLAT as distant from the first template, resulting in chromosomal rearrangements and typically fusions with other chromosomes (Supplementary Data S14). For example, one MMB-TI event that mediated a fusion between Chr 7 and Chr X (LT-1) (Figure 4E) contained 4bp of microhomology at its first junction, copied 19bp from Chr7 and using 9bp of microhomology, proceeded to Chromosome X. These junctions could be minimally extended as microhomeology with only a single base mismatch or gap. The second MMB-TI event (LT-2) that mediated a fusion between Chromosome 7 and Chromosome 6 contained 5bp (extended to 7bp of microhomeology), copied 26bp from Chromosome 7, and used 5bp of microhomology to continue to

Chromosome 6 (Figure 4F). Interestingly, additional matching bases were found further behind 3 of the 4 junctions in these examples (Figure 4E, F – yellow boxes).

Description of Clonal MMB-TI events from lung adenocarcinoma patient

To analyze MMB-TI events with higher clonality, we focused on MMB-TIs called by MMBSearch with more than 3 non-duplicate reads and identified two events, J2 and J6, that were detected by 14 and 11 reads respectively (Figure 6; Supplementary Data S18). Importantly, in addition to an initial MMB-TI copied from a nearby template, both of these junctions also included a second region copied from a distant template, resulting in chromosomal rearrangements (Figure 6Ai, iii, Bi, iii). Using BLAST, we confirmed the locations of the secondary templates and designed primers to span the rearrangements (Supplementary Data S20). We confirmed the rearrangements first by PCR and second by Sanger sequencing of the amplified fragments to verify their exact structure (Figure 6Aii, Bii and Figure 7C).

The J2 MMB-TI created a fusion between chromosomes 5 and 20 (Figure 6Ai; Supplementary Data S18). Analysis of the junctions suggested that this event likely initiated on chromosome 20, copied 47 bp from a nearby template, and subsequently switched to chromosome 5, leading to a translocation (Figure 6Aiii). The first junction of this MMB-TI mutation did not contain a matching base at the 3’ end itself, but 3 bp directly adjacent to it could act as microhomeology to mediate the template switch. The right junction that mediated the translocation contained 7 matching bases beginning from the 3’ end and gained 2 additional bases when allowing for microhomeology (Figure 6Aii).

The J6 MMB-TI event created a chromosomal rearrangement that fused two regions of chromosome 5 located 34 kb away from one another (Figure 6Bi; Supplementary Data S18)). Analysis of the junctions suggested that this event likely initiated from the region telomere-proximal to the fusion, copied 65 bp from a nearby template, and then switched to the centromere-proximal junction (Figure 6Biii). The centromere-proximal junction contained 2 bp of microhomology that could be extended to 3 bp of microhomeology if 1 gap is allowed. The

telomere-proximal junction only contained 1 bp of microhomology, and likewise 2 bp of microhomeology (Figure 6Bii J6).

In looking for additional junctions nearby to those found on chromosomes 5 and 20 with similar levels of read evidence that might be related to the MMB-TI events described above, we searched the clusters of reads generated by MMBSearch and found 7 additional breakpoint junctions that were detected between 9 and 15 reads each. We also confirmed these junctions by PCR (Figure 7A, C; Supplementary Data S18, S19). All of these similarly evidenced junctions also contained varying lengths of microhomology (Figure 7B; Supplementary Data S18, S19). While these breakpoints are not sufficiently clonal to produce a change in ploidy, the increased levels of reads associated with them implies a sub-clonal increase in copy number (Supplementary Figure 9A, B). Analysis of clusters also revealed that the cluster containing MMB-TI event J6 contained a second junction, J5. The first breakpoint for J6 and the J5 breakpoint were less than 100 bp from each other (Figure 6Bii, iii), and this proximity strongly suggests a single breakage event. Importantly, this implies that the MMB-TI event found at J6 was the initiating template switching event that led to discoordination of two ends from a single break, resulting in two distinct rearrangements at a single locus. Because the J6 MMB-TI event replaces the majority of its template, we can speculate that it was copied from either the lagging strand in the context of a stalled replication fork (Figure 7Di), or from the top strand within the BIR D-loop (Figure 7Dii), before template switching to a more distal template, while the J5 junction forms as the result of template switching initiated by a second broken end (Figure 7Di, ii).

In addition to events that did not produce a change of ploidy, Chromosomes 5 and 20 in the tumor also displayed 3n regions (Supplementary Figure 9A) implying that the breakpoints of those 3n regions were fully clonal, and likely existed starting from the point the tumor was established. We investigated these fully clonal rearrangement events and found a similar pattern of rearrangements and coincident breakpoints, one of which again contained an MMB-TI event on one of two coincident junctions just 63 bp away from each other (Supplementary Data S18, Junctions J14 and J15).

Analysis of fibroblast and cultured GM1604 cells with MMBSearch set to low clustering threshold

We reanalyzed one fibroblast sample from (1), with identical parameters to those used to call MMB-TI events in the lung tumor sample that we sequenced (BO13-UIBB-1654). The analysis found only 22 MMBSearch calls unique to Chr1 in the fibroblast (Supplemental Figure 8A) and only 3 of the calls appeared to match the MMB-TI signature when manually inspected, while the remaining were either microsatellites or alignment errors. It is important to note that the fibroblast sample from (1) was prepared using a comparable library preparation kit to that which was used to prepare our lung tumor sample, except that it included a short PCR amplification step prior to sequencing. Our lung tumor sample by contrast was prepared using a PCR-free library prep kit to avoid accumulating palindromic artifacts introduced during end-blunting which have been shown to be preferentially amplified during library construction that utilizes PCR in highly fragmented samples (3). Together, the MMB-TI events called sub-clonally in the fibroblast sample were extremely rare.

As a secondary control, we performed sequencing on cultured GM1604 human fetal lung fibroblast cells. We chose this cell line as a control because it is non-cancer and originates from the lung. We selected single cells from the cultures and grew clonal lineages prior to sequencing (See Materials and Methods for culturing details). These cultured cells were sequenced using the same PCR-free library preparation as the lung tumor sample, and the same sequencing method (see Materials and Methods for details). We analyzed the sequencing reads from the GM1604 clones with MMBSearch and found that on Chr1 there was a low level (2.5-5%) of calls that matched the MMB-TI signature (Supplementary Figure 8B). Together we conclude that the number of MMB-TI insertions observed in both fibroblasts and the GM1604 clones was extremely low as compared to what was observed in both our sequencing of IB lung tumor, and IV lung tumor sequencing obtained from dbGaP (Supplementary Figure 8A), supporting our idea that the high quantity of MMB-TI events found in the lung tumor samples is related to the pathology of the tumor and not a technical artifact.

References.

1. Saini, N., Roberts, S.A., Klimczak, L.J., Chan, K., Grimm, S.A., Dai, S., Fargo, D.C., Boyer, J.C., Kaufmann, W.K., Taylor, J.A. *et al.* (2016) The Impact of Environmental and Endogenous Damage on Somatic Mutation Load in Human Skin Fibroblasts. *PLoS Genet*, **12**, e1006385.
2. Huang, W., Li, L., Myers, J.R. and Marth, G.T. (2012) ART: a next-generation sequencing read simulator. *Bioinformatics*, **28**, 593-594.
3. Star, B., Nederbragt, A.J., Hansen, M.H., Skage, M., Gilfillan, G.D., Bradbury, I.R., Pampoulie, C., Stenseth, N.C., Jakobsen, K.S. and Jentoft, S. (2014) Palindromic sequence artifacts generated during next generation sequencing library preparation from historic and ancient DNA. *PLoS One*, **9**, e89676.

Supplementary Figure 1

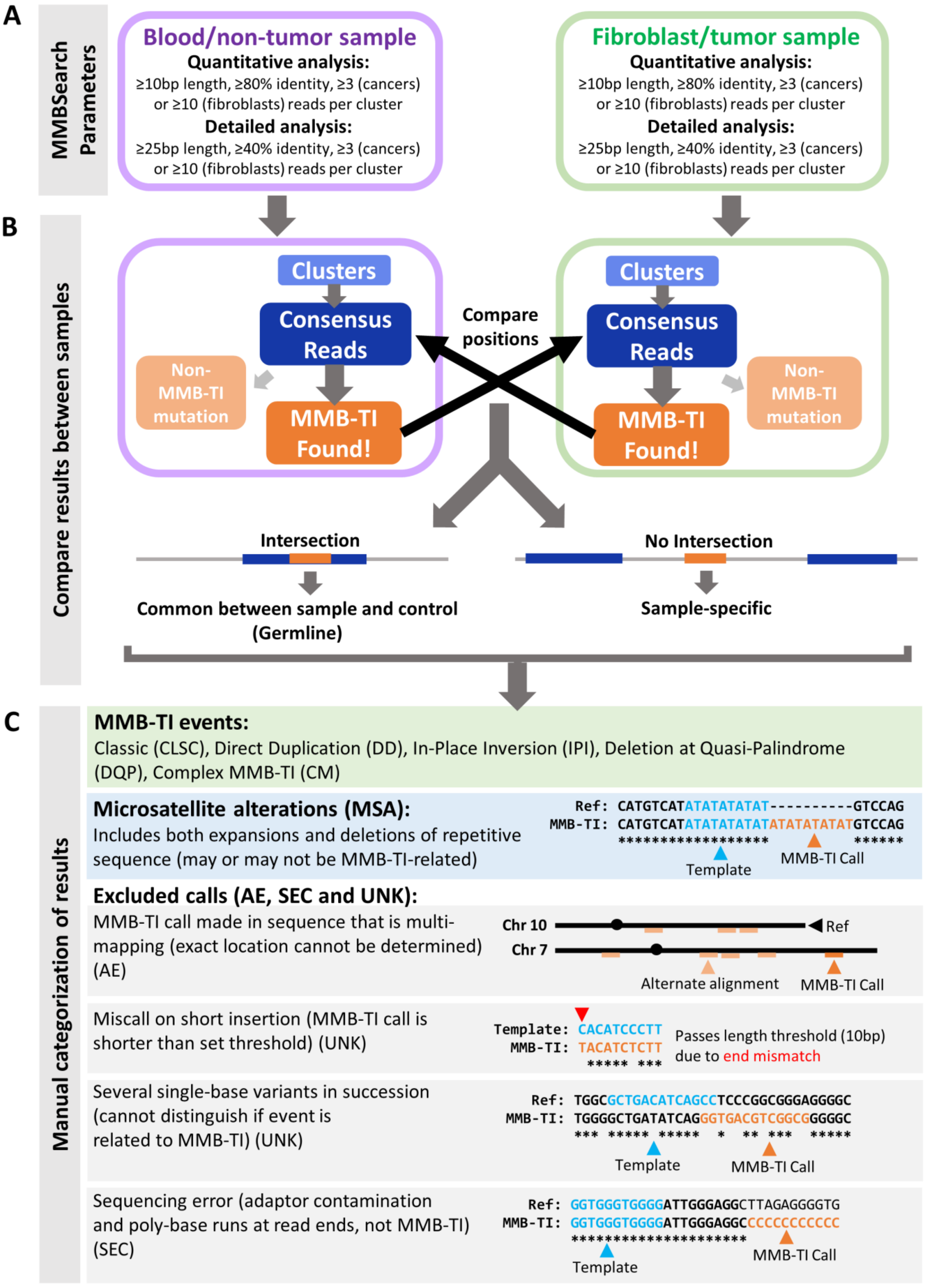

Supplementary Figure 2

A

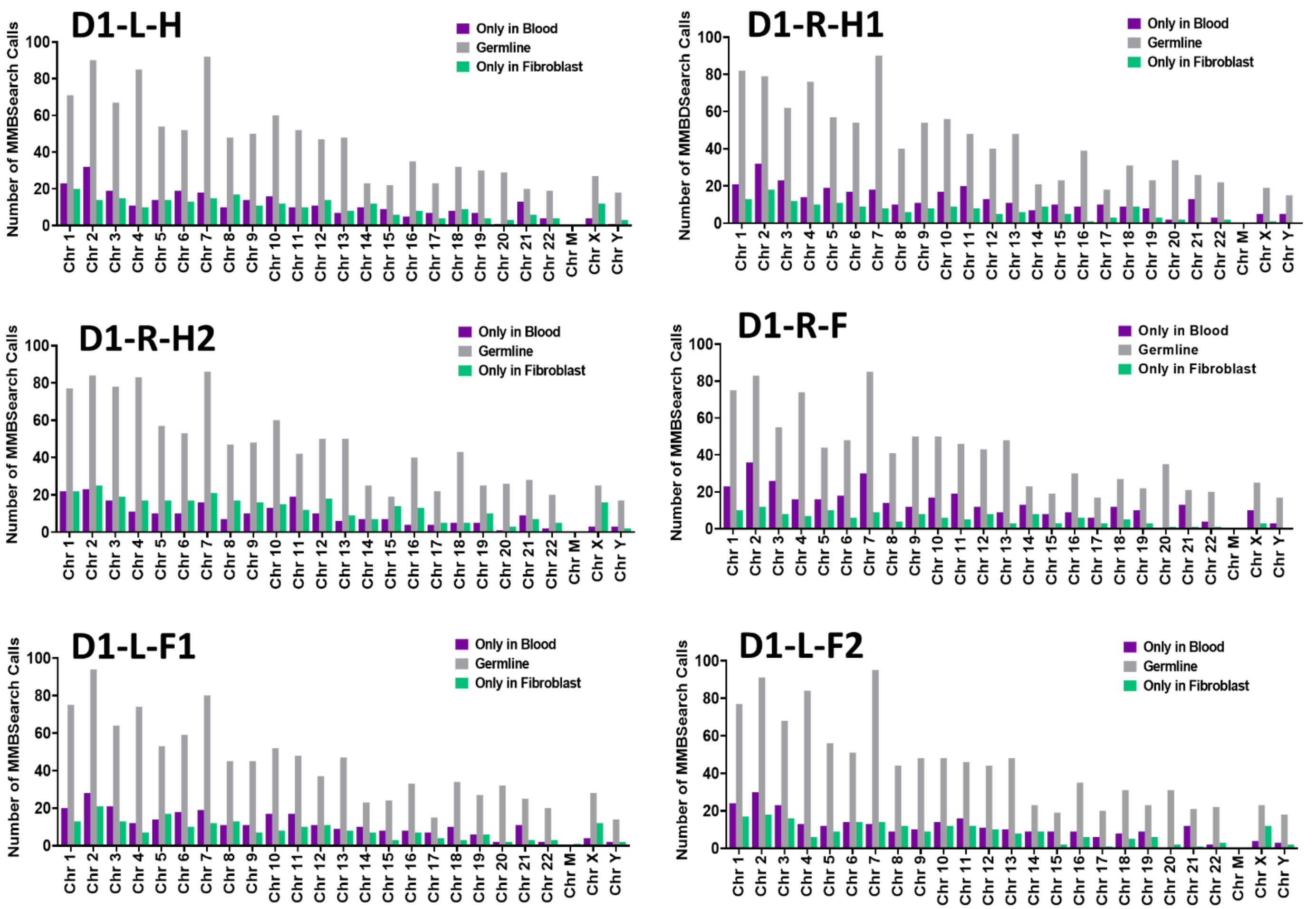

B

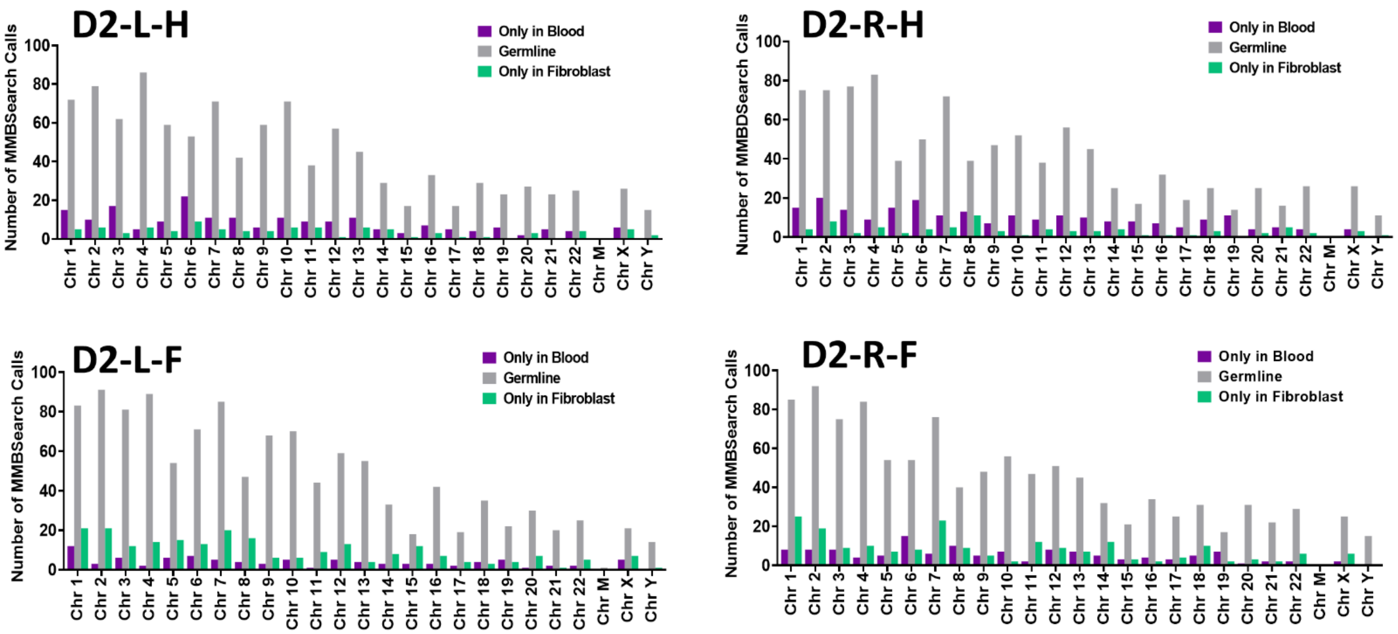

Supplementary Figure 3

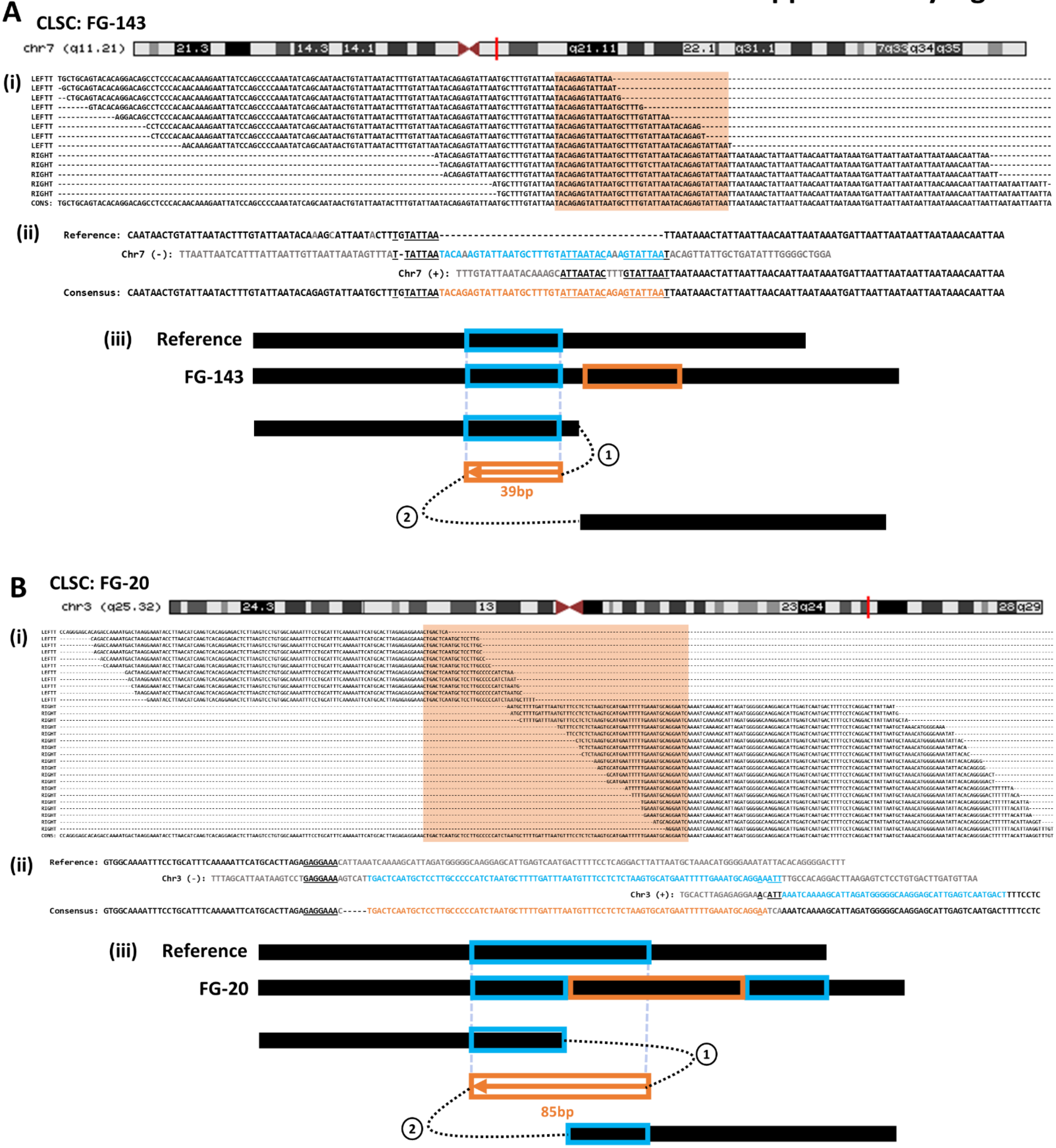

Supplementary Figure 4

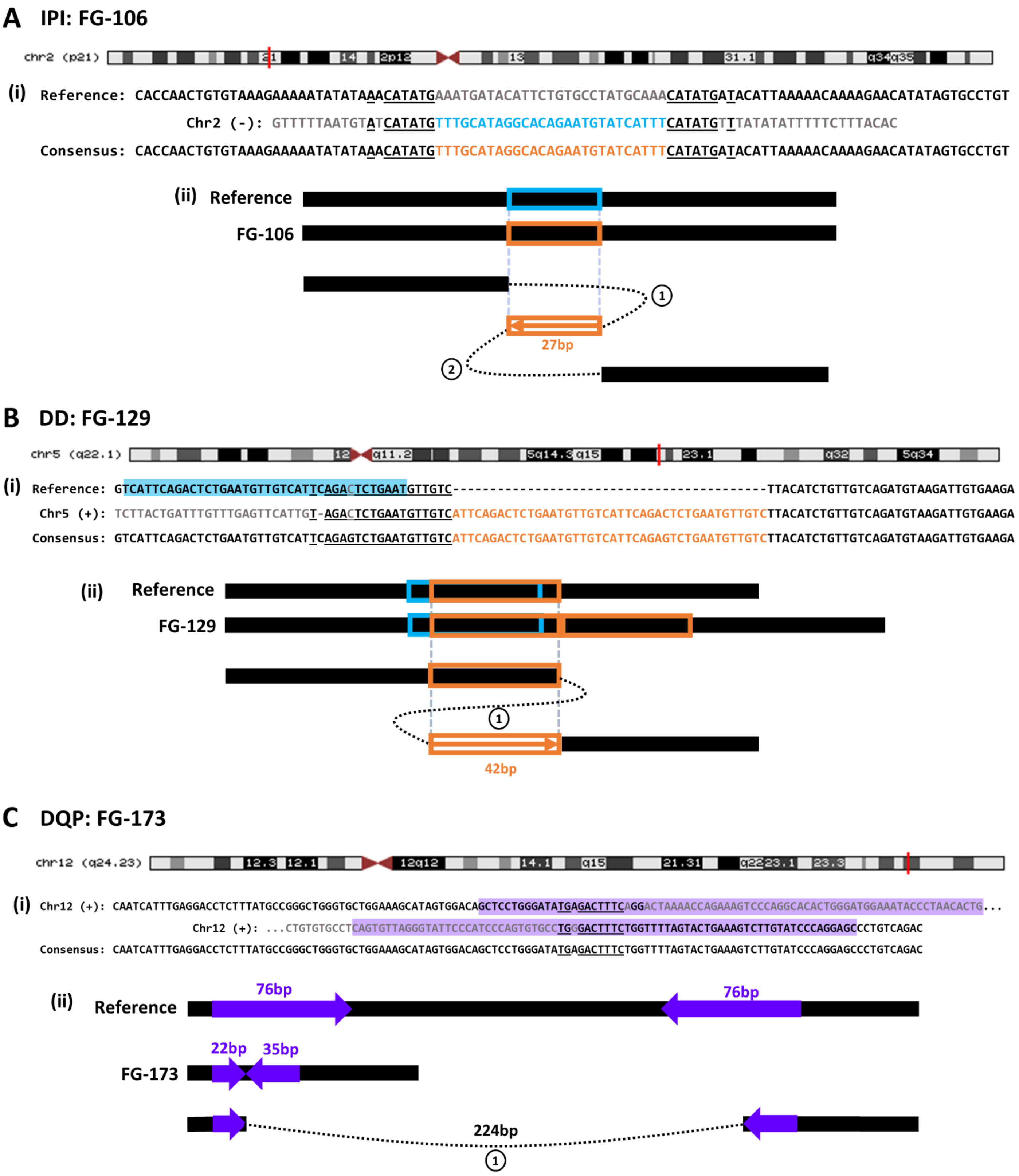

Supplementary Figure 5

A

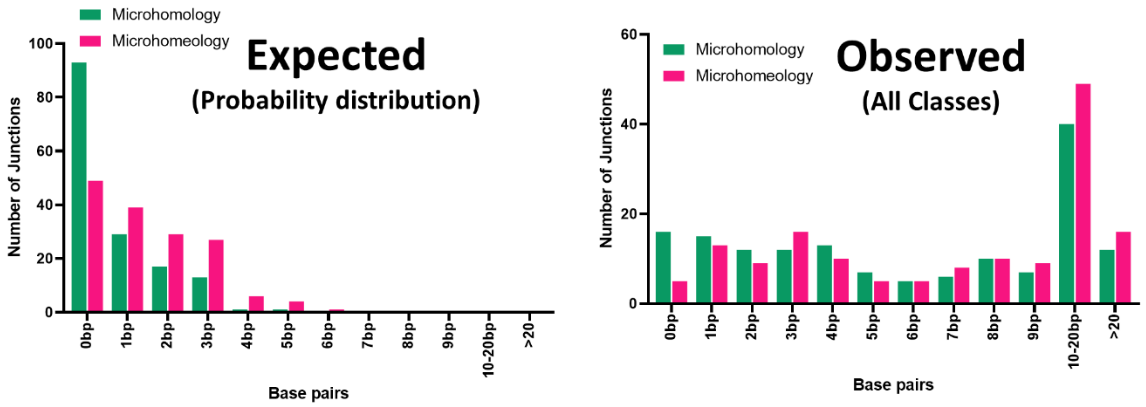

B

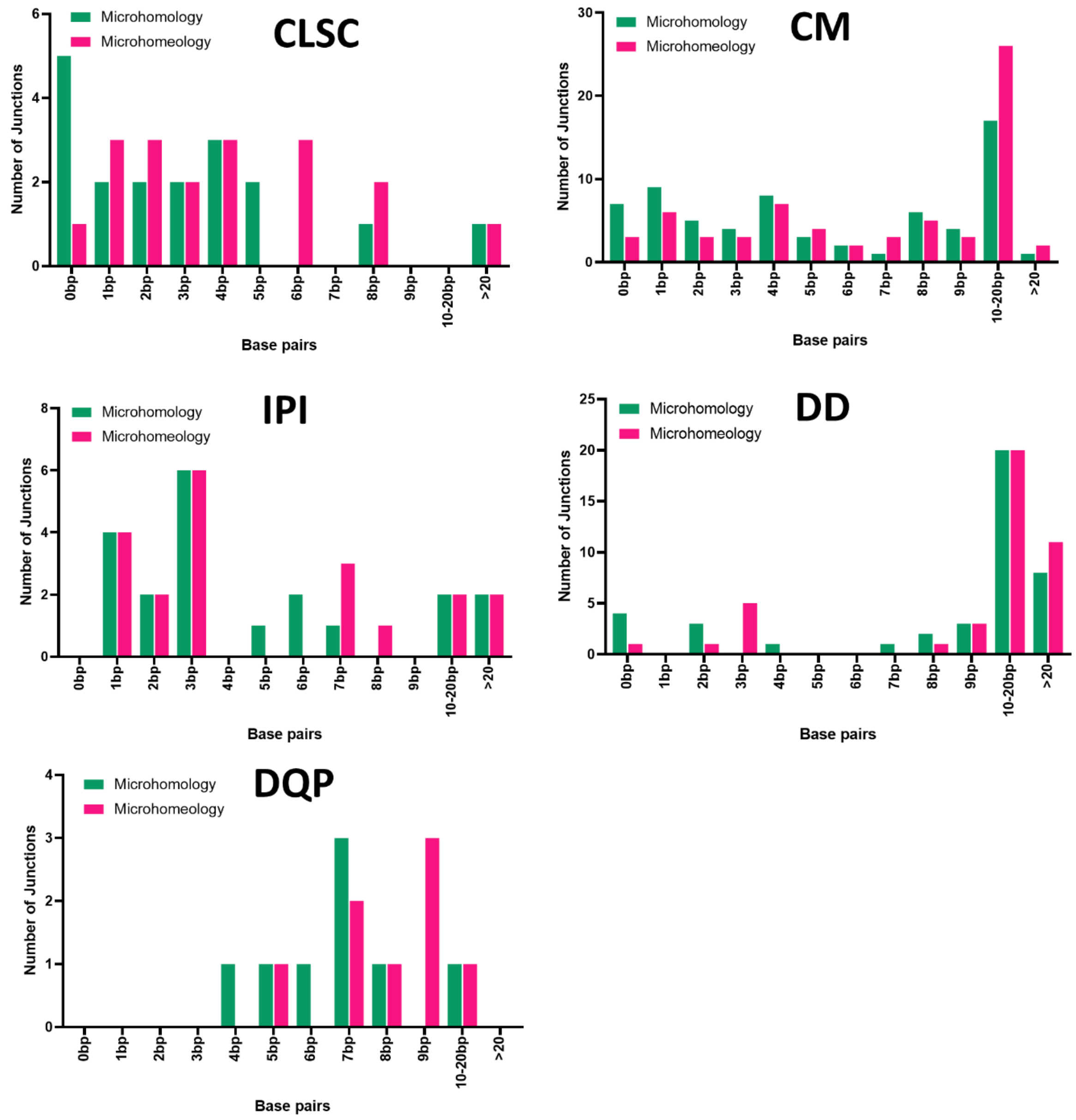

Supplementary Figure 6

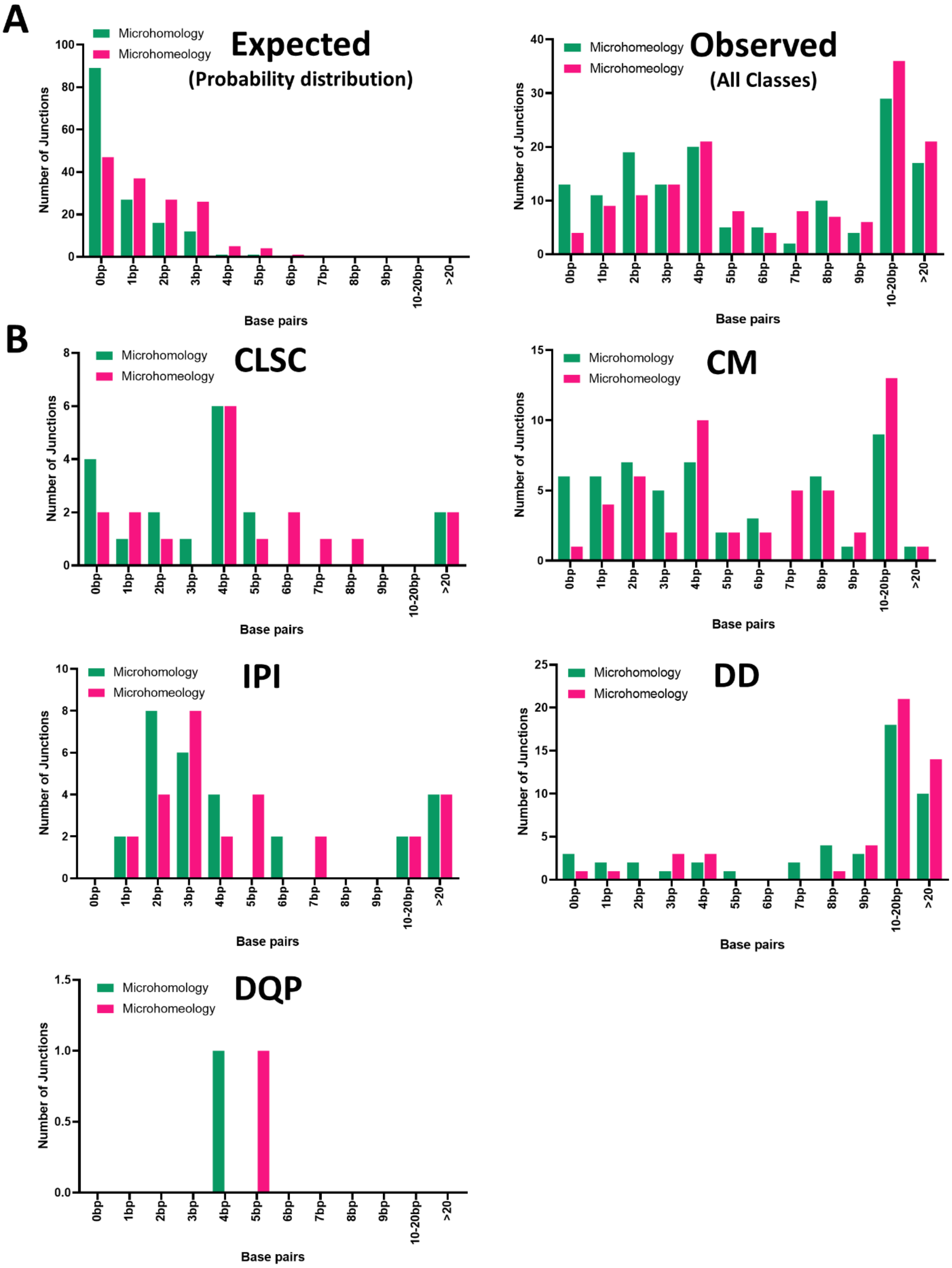

Supplementary Figure 7

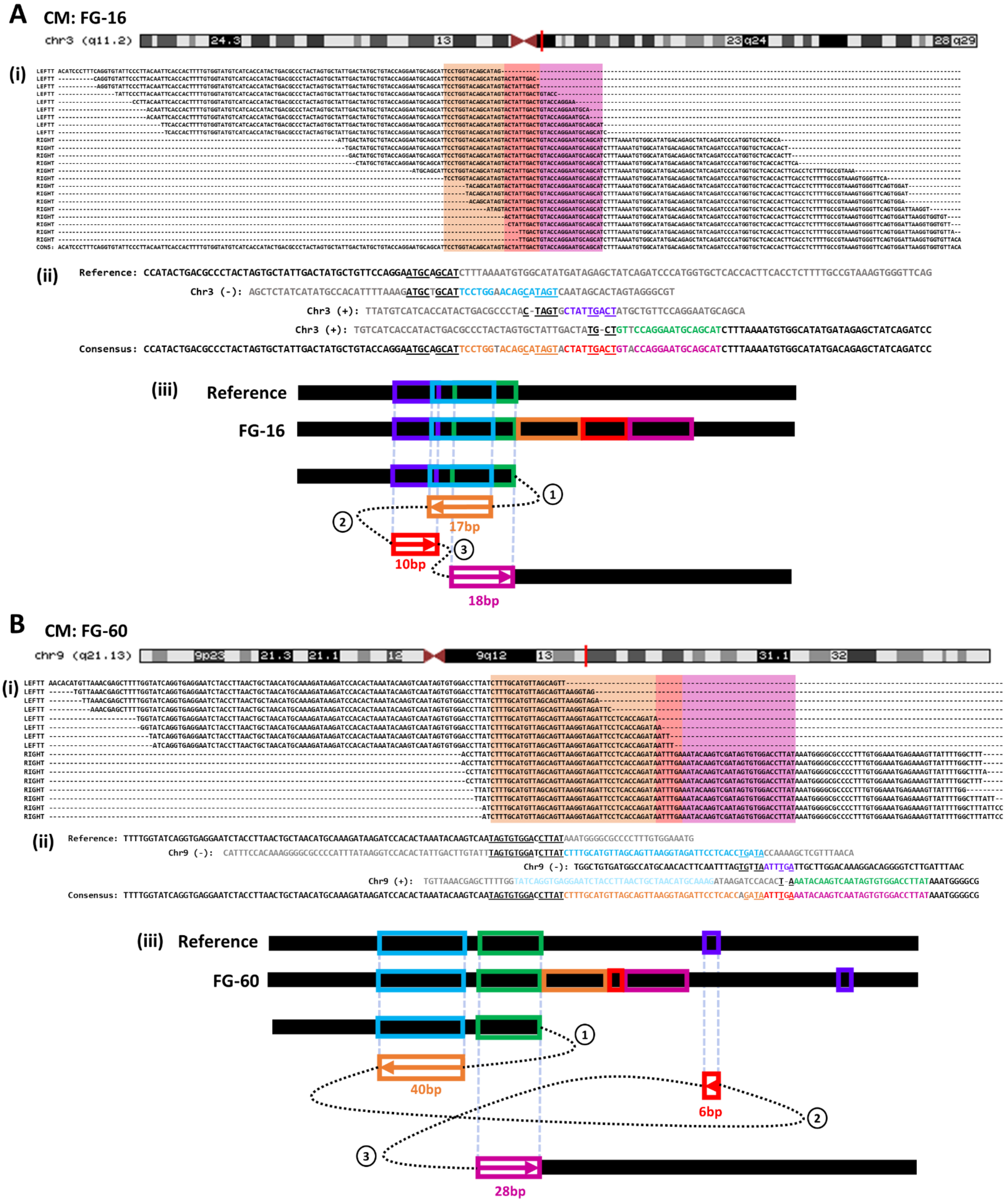

Supplementary Figure 8

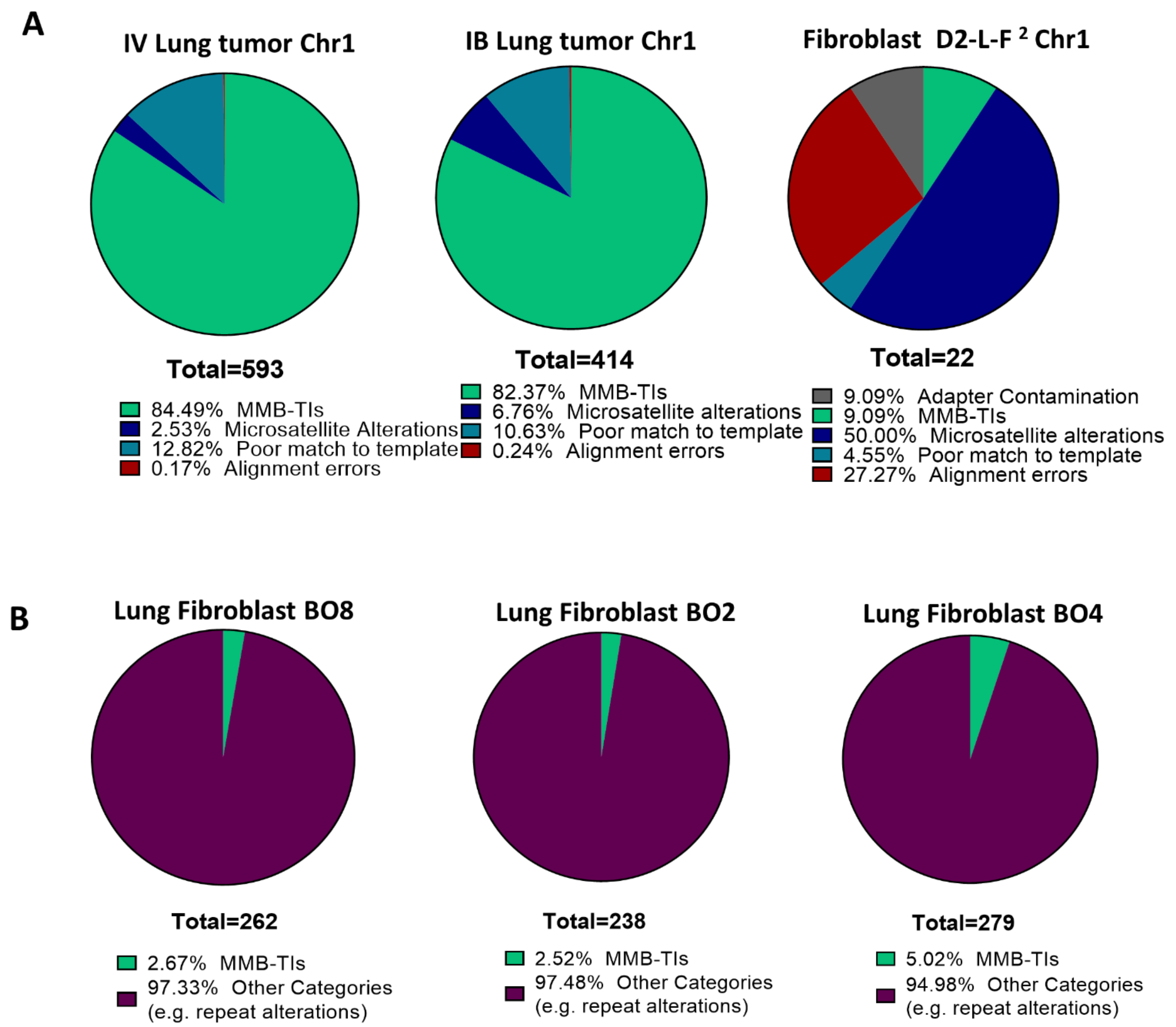

Supplementary Figure 9

A

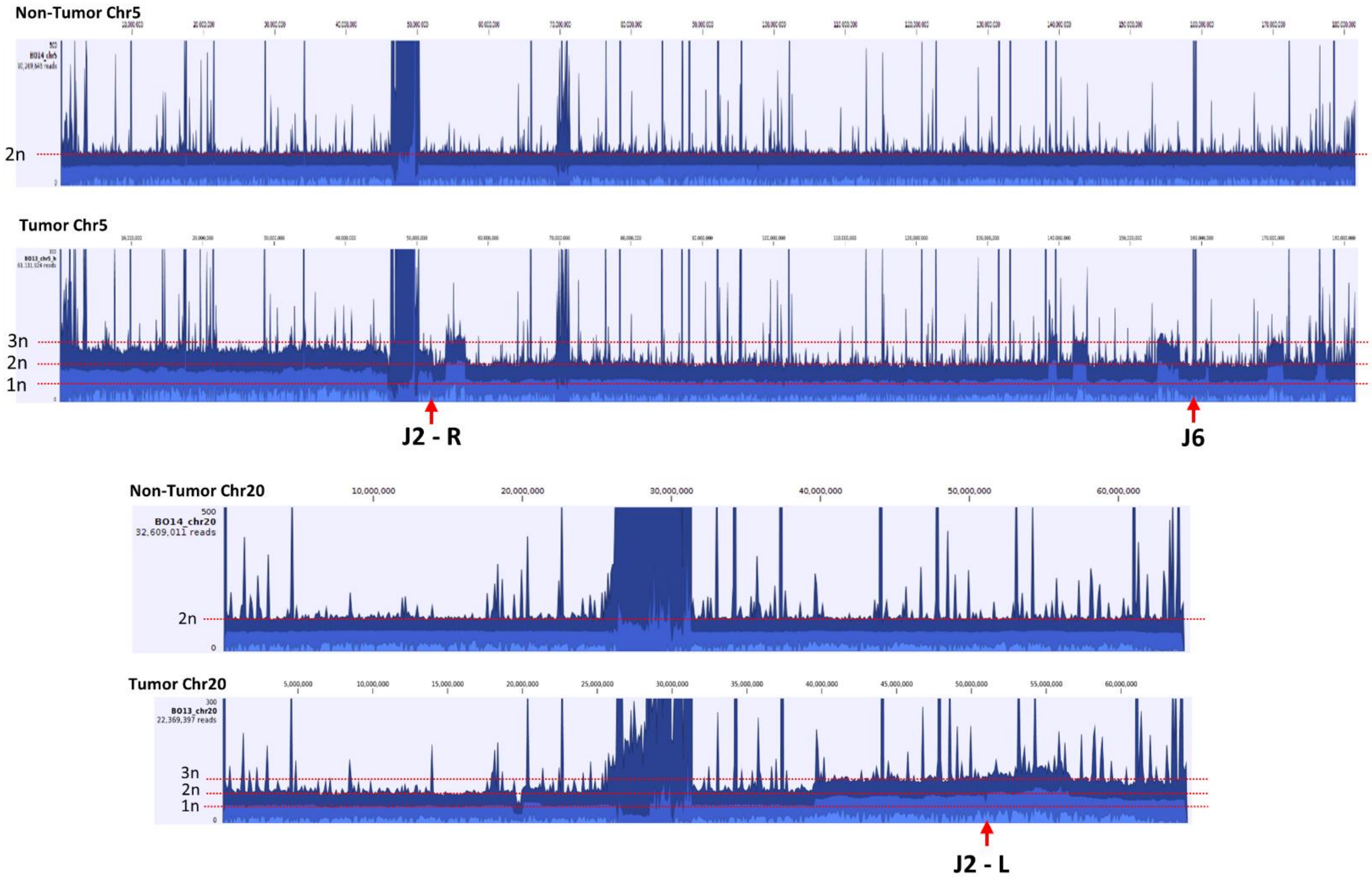

B

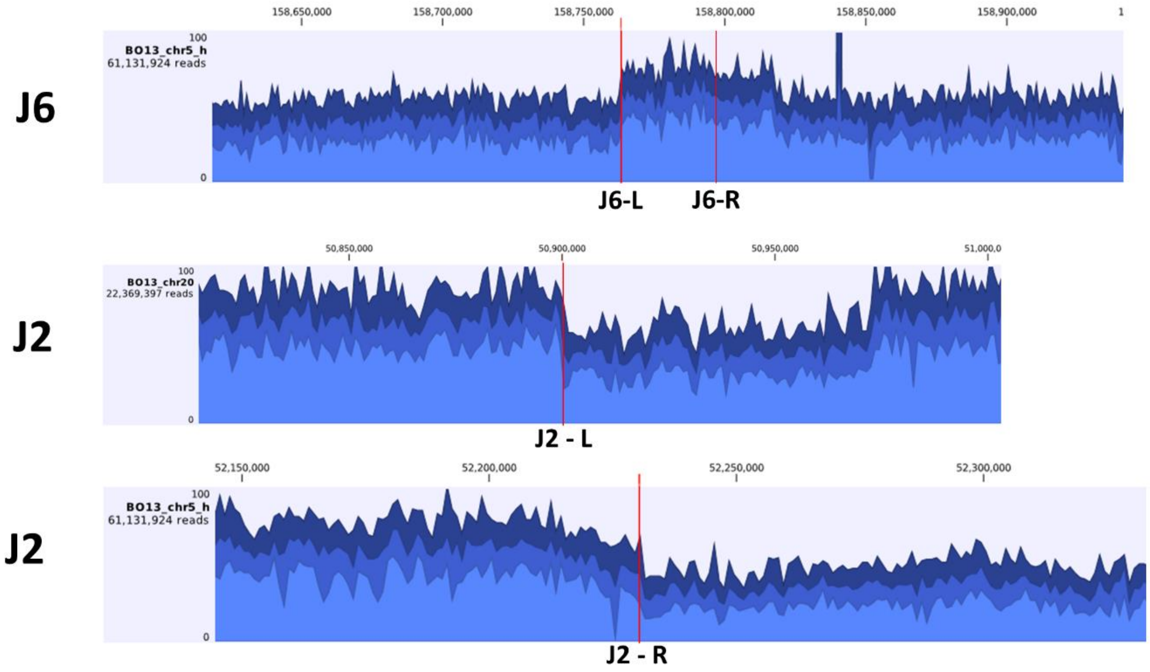

Supplementary Data S1: Summary of MMBSearch calls from 10 clonal fibroblast lineages of 2 individuals

	All MMBSearch calls †		All Fibroblast-specific MMBSearch calls ‡			
**Fibroblast clone ID ***	**Germline calls**	**Fibroblast-specific calls**	**Alignment and adapter errors**	**Microsatellite alterations**	**Multi-clone MMB-TIs**	**Clone-specific MMB-TIs**
**D1-L-H**	1094 (82%)	244 (18%)	35 (14%)	186 (76%)	23 (9%)	0 (0%)
**D1-R-H1**	1057 (87%)	158 (13%)	29 (18%)	117 (74%)	12 (8%)	0 (0%)
**D1-R-H2**	1048 (84%)	203 (16%)	31 (15%)	151 (74%)	21 (10%)	0 (0%)
**D1-L-F1**	1105 (78%)	312 (22%)	55 (18%)	231 (74%)	25 (8%)	1 (0.3%)
**D1-L-F2**	1066 (84%)	206 (16%)	18 (9%)	164 (80%)	24 (12%)	0 (0%)
**D1-R-F**	998 (88%)	130 (12%)	25 (19%)	92 (71%)	13 (10%)	0 (0%)
**D2-L-H**	1058 (92%)	90 (8%)	22 (24%)	63 (70%)	5 (6%)	0 (0%)
**D2-R-H**	984 (93%)	78 (7%)	17 (22%)	56 (72%)	5 (6%)	0 (0%)
**D2-L-F**	1089 (85%)	195 (15%)	50 (26%)	136 (70%)	9 (5%)	0 (0%)
**D2-R-F**	1177 (84%)	229 (16%)	63 (28%)	162 (71%)	4 (2%)	0 (0%)

***** Fibroblasts were derived from individual 1 (D1) and individual 2 (D2) from the study of [1]. Clone sites: left hip (D1-L-H, D2-L-H), Right hip (D1-R-H1, D1-R-H2, D2-R-H), left forearm (D1-L-F1, D1-L-F2, D2-L-F) and right forearm (D1-R-F, D2-R-F).
† MMBSearch calls from clusters with ≥10 reads, insertions that are ≥10bp in length, and ≥80% identity between template and insertion.
‡ Percentages are out of total fibroblast-specific calls.
